# Assessing effects from four years of industry-led badger culling in England on the incidence of bovine tuberculosis in cattle, 2013–2017

**DOI:** 10.1038/s41598-019-49957-6

**Published:** 2019-10-11

**Authors:** Sara H. Downs, Alison Prosser, Adam Ashton, Stuart Ashfield, Lucy A. Brunton, Adam Brouwer, Paul Upton, Andrew Robertson, Christl A. Donnelly, Jessica E. Parry

**Affiliations:** 1 Department of Epidemiological Sciences, Animal and Plant Health Agency, Woodham Lane, New Haw, Surrey, KT15 3NB UK; 2 Veterinary Epidemiology, Economics and Public Health Group, The Royal Veterinary College, Hawkshead Lane, Hatfield, Hertfordshire, AL9 7TA UK; 3 Environment and Sustainability Institute, University of Exeter, Penryn, Cornwall, TR10 9FE UK; 4 National Wildlife Management Centre, Animal and Plant Health Agency, Woodchester Park, Nympsfield, Gloucestershire, GL10 3UJ UK; 5 MRC Centre for Global Infectious Disease Analysis, Department of Infectious Disease Epidemiology, Faculty of Medicine, School of Public Health, Imperial College London, St Mary’s campus, Norfolk Place, London, W2 1PG UK; 6 Department of Statistics, University of Oxford, 24-29 St Giles’, Oxford, OX1 3LB UK

**Keywords:** Epidemiology, Outcomes research

## Abstract

The objective was to measure the association between badger culling and bovine tuberculosis (TB) incidents in cattle herds in three areas of England between 2013–2017 (Gloucestershire and Somerset) and 2015–2017 (Dorset). Farming industry-selected licensed culling areas were matched to comparison areas. A TB incident was detection of new *Mycobacterium bovis* infection (post-mortem confirmed) in at least one animal in a herd. Intervention and comparison area incidence rates were compared in central zones where culling was conducted and surrounding buffer zones, through multivariable Poisson regression analyses. Central zone incidence rates in Gloucestershire (Incidence rate ratio (IRR) 0.34 (95% CI 0.29 to 0.39, p < 0.001) and Somerset (IRR 0.63 (95% CI 0.58 to 0.69, p < 0.001) were lower and no different in Dorset (IRR 1.10, 95% CI 0.96 to 1.27, p = 0.168) than comparison central zone rates. The buffer zone incidence rate was lower for Gloucestershire (IRR 0.64, 95% CI 0.58 to 0.70, p < 0.001), no different for Somerset (IRR 0.97, 95% CI 0.80 to 1.16, p = 0.767) and lower for Dorset (IRR 0.45, 95% CI 0.37 to 0.54, p < 0.001) than comparison buffer zone rates. Industry-led culling was associated with reductions in cattle TB incidence rates after four years but there were variations in effects between areas.

## Introduction

Industry-led badger culling as a policy to control bovine tuberculosis (TB) in cattle was introduced by the Department for Environment, Food & Rural Affairs in England in 2013. The policy has been conducted predominantly in locations within the high risk area (HRA) for TB. Between 2013 and 2017, the HRA covered 39,175 km^2^ and included all or part of 15 counties extending from Cornwall in the southwest to Derbyshire in the midlands plus East Sussex. Since 2011, *Mycobacterium bovis* (the bacterium that causes bovine TB) infection has been detected at least 3000 HRA cattle herds annually. In 2017, the percentage of cattle herds within HRA counties with new TB infection detected, ranged between seven and 23%; a county level incidence rate ranging between seven and 23 TB incidents per 100 herd years at risk (HYR)^1^.

Defra aims to achieve Officially Tuberculosis Free (OTF) status for England by 2038, and the strategy to achieve this includes control of TB in badgers^2^. Both cattle and the European badger (*Meles meles*) are reservoirs for *M. bovis* and there is evidence that culling badgers can reduce TB in cattle^3–6^. Culling areas have been selected by the farming industry, although they must meet Government licensing criteria^7^. In 2013, licence criteria required that the application area be greater than 150 km^2^ with at least 70% of that land accessible for culling^8^. Additionally, a reduction of at least 70% of the estimated badger population had to be planned for the first year of culling, an effective cull conducted for a minimum of four years and farms in the area were required to have reasonable biosecurity in place.

Culling licence criteria were informed by results from the Randomised Badger Culling Trial (RBCT) conducted between 1998 and 2006^9^. The RBCT showed a statistically significant decrease in confirmed TB incidence (where *M. bovis* has been detected through post-mortem tests in at least one animal from each herd) of 23% (95% Confidence Interval (CI) 12 to 33%, p < 0.001) over four years in 100 km^2^ culled areas compared to non-culled areas^6,10^. An increase of 25% (95% CI 1 to 56%, p = 0.057) in confirmed TB was also observed on two km wide land surrounding the culled areas relative to land surrounding non-culled areas, during the period culling was conducted^10^.

Licences for culling have been issued for two areas (in Gloucestershire and Somerset) from 2013, one area (in Dorset) from 2015, seven areas from 2016 and 11 areas in both 2017 and 2018. All areas are located within the HRA except one area in 2018, which is located in the Low Risk Area for TB in England. The culling areas are larger than the RBCT areas and also differ in a number of other important ways. For example, culling during the RBCT was carried out by government and involved cage-trapping badgers; with the whole area simultaneously trapped over a period of approximately 10 days. In contrast, current badger culls are carried out by the farming industry, and involve a combination of cage trapping and controlled shooting (where free roaming badgers are shot at night), with effort spread over a period of six weeks or more. Consequently, it is unclear whether current badger culls would produce similar changes in cattle TB incidence to those observed during the RBCT.

The Animal and Plant Health Agency (APHA) has been commissioned to monitor and evaluate the effects of culling on TB incidence in cattle herds in culling areas. The analysis of effects in cattle herds over two years since badger culling began in Gloucestershire and Somerset has already been reported^11^. This analysis showed a statistically significant decrease in Officially Tuberculosis Free Withdrawn (OTF-W) incidence in both culled areas compared to areas with no culling. An OTF-W incident is a new outbreak of TB in a herd disclosed by field testing or slaughterhouse surveillance and where *M. bovis* is detected through post-mortem tests in at least one animal in the herd. The OTF-W incidence rate ratio (IRR) was 0.79 for Somerset (95% CI 0.72 to 0.87) and 0.42 for Gloucestershire (95% CI 0.34 to 0.51) after adjustment for confounding factors. An increase in incidence was observed in the two km buffer zone around the Somerset culled area (IRR 1.38, 95% CI 1.09 to 1.75) but not in Gloucestershire (IRR 0.91 95% CI 0.77 to 1.07).

The statistically significant decrease in cattle TB incidence associated with culling in Gloucestershire and Somerset over two years was unexpected given the statistical power of the study^12^. The aims of this new analysis is to determine firstly whether a decrease in TB incidence rates in cattle associated with culling has been sustained in Gloucestershire and Somerset; secondly whether a similar effect can be detected after two years in Dorset; and thirdly whether an effect on TB incidence can be detected in two km wide buffer zones surrounding culled areas. The null hypothesis being tested is that TB incidence is the same in the intervention areas (where culling has been conducted) and their comparison areas in the years since badger culling began^11^.

## Methods

### Intervention area coverage, cohort of herds exposed to culling, follow-up period

**Figure 1:**
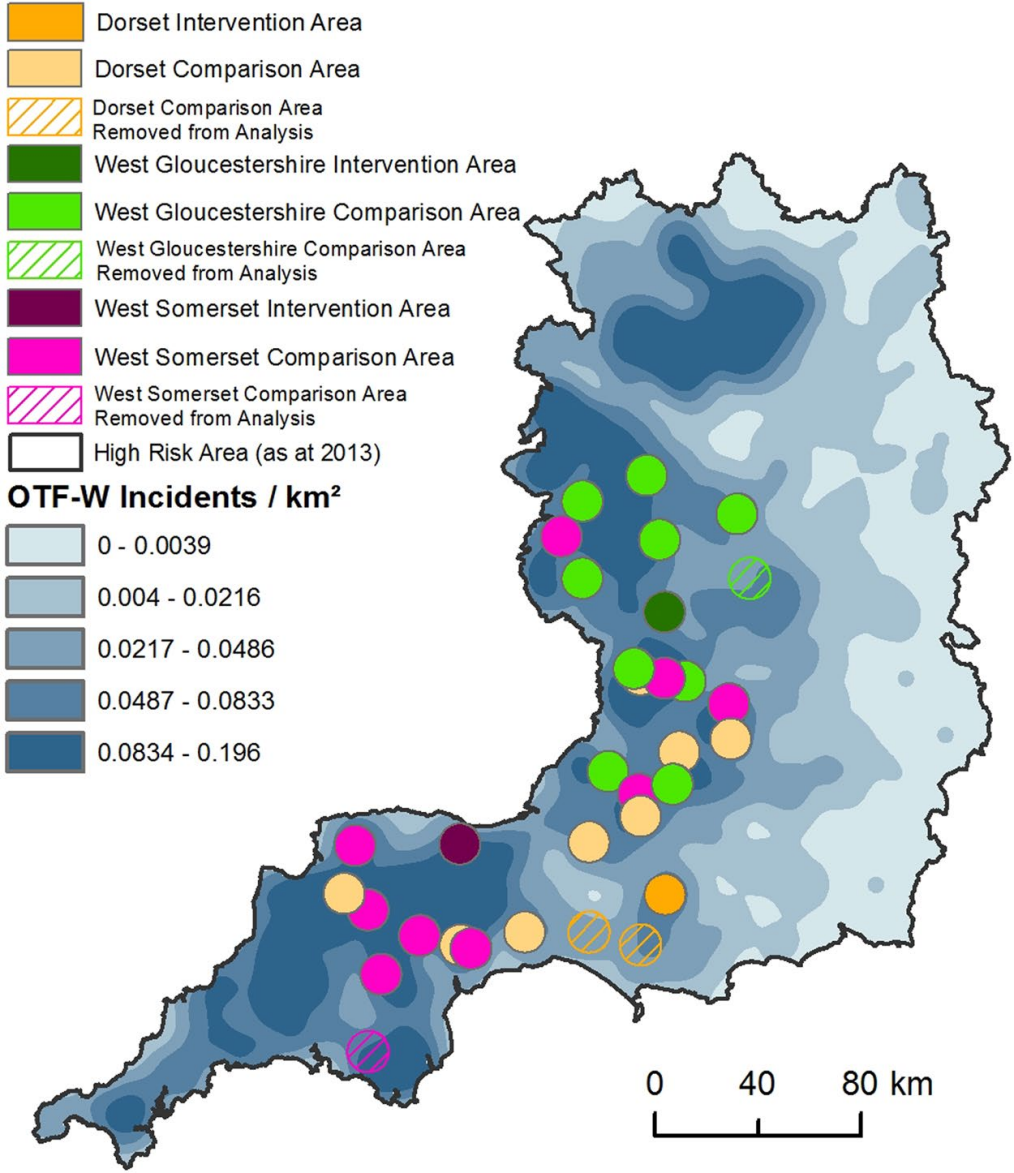
Location of Gloucestershire, Somerset and Dorset intervention areas licensed in 2013 and 2015 and comparison areas. Culling and comparison areas are symbolised by solid circles. OTF-W is Officially Tuberculosis Free Withdrawn, which is where *M. bovis* has been detected through post-mortem tests in at least one animal in the herd. OTF-W incident density is from 2013 and was created using the spatial analyst kernel density tool within ArcGIS 10.0. Boundaries to the High Risk Area and Edge Area are those that were in place in 2013. Comparison areas removed from analysis due to overlapping intervention areas licensed in 2015 and 2016 are shown as hatched circles. Circles show the location of intervention and comparison areas but do not approximate actual shape and size.

The intervention areas in Gloucestershire, Somerset and Dorset selected by industry are 311, 256 and 223 km^2^ in size respectively and located in the HRA of England (Fig. 1). Culling took place during a six-week period each autumn in each area. In this analysis, the culling areas are referred to as the central zones. Each central zone has been allocated a two km wide buffer zone from its boundary using ArcGIS (ESRI Releases 10.0–10.3 Redlands, CA, USA). Cattle herds in existence in the APHA surveillance database (Sam) in the central and buffer zones when culling started (the baseline date, Table S1) formed the cohort of herds exposed to badger culling and monitored for changes in TB incidence^11^. The follow-up period in this analysis was four years from autumn 2013 to autumn 2017 in Gloucestershire and Somerset and two years from autumn 2015 to autumn 2017 in Dorset (Table S1).

### Selection of comparison areas

The methodology and criteria for the selection of comparison areas for the Gloucestershire and Somerset intervention areas have been reported in detail in Brunton *et al*.^11^. The same comparison areas for Gloucestershire and Somerset are used in the current analysis. Comparison areas for Dorset, which was licenced two years later, have been selected for the current analysis using the methodology reported previously^11^. Geographical Information System programming was used to generate a population of potential comparison areas within the HRA of the same size and shape as the Dorset area. Each area was ranked by degree of similarity to the Dorset area on the basis of the number of OTF-W incidents in the previous three years, the number of herds in the area, the median herd size, the proportion of the area (if any) that was in a RBCT proactive cull area and distance to the Dorset intervention area. The 10 areas that most closely matched the Dorset intervention area and did not contain land already within Gloucestershire or Somerset intervention areas or land within two km of any intervention area boundary (at the time of the baseline date) were selected. Cattle herds in existence in comparison area central and buffer zones on the baseline date formed the cohort of herds compared to herds in the intervention area central and buffer zones.

### Main outcome variable and time at risk

An area level analysis was conducted. The main outcome variable was the OTF-W incidence rate in cattle herds for each area. Effects were also estimated for all TB incidents (OTF-W plus OTFS (Officially Tuberculosis Free Suspended)), where reactors to the Single Intradermal Comparative Cervical Tuberculin (SICCT) test had been detected in a herd but infection had not been confirmed by post-mortem tests. Herd years at risk (HYR) were calculated using results from whole herd tests as the sum of the time cohort herds were unrestricted and therefore at risk of new infection (a new TB incident) during the period of interest^13^. HYR data used previously^11^ were updated to remove implausible values for time at risk. This meant that the sum of time at risk for all herds in an area as opposed to the median estimate of time at risk could be used thereby bringing the methodology in line with national statistics reporting^14^. Herds could move out of an area during the follow-up period because either the farmer moved the herd off the land in the area to which the farm was originally assigned to land outside of that area, or the original geo-reference for the location of the herd was found to be incorrect and the subsequent correction placed the herd outside of the area. Herds that moved out of an area but were still in existence during the follow-up period contributed incidents and HYR for the calculation of area-level incidence rates for the area in which they were originally located on the baseline date. Similar to an “intention to treat” analysis, the purpose was to reduce any bias due to differences in the number of herds leaving areas between intervention and comparison areas.

### Confounding factors

Comparison areas, although selected based on their similarity to intervention areas will vary to a greater and lesser extent from the intervention areas in factors associated with TB incidence in cattle^14,15^. Factors (attributes of the herds and the area) that could be associated with TB incidence and with selection of an area for badger culling were extracted from the APHA cattle surveillance database and other sources and summarised for each area. These factors included all those in Brunton *et al*.^11^ plus a new variable describing badger density that included new survey data^16,17^ (see Supplement). As a result of data checks, corrections were made to two variables used previously; to the proportion of land in flood zone 3 and the proportion of land that had been in a proactive area of the RBCT.

### Overlap between areas

Early in the project design phase, it was apparent that comparison areas could be overlapped by intervention areas licensed in later years and that comparison areas to different intervention areas might also overlap^18^. Rules were developed, prior to any analyses, to address allocation of herds between overlapping areas and for summarising confounder data for overlapping areas (Fig. S1, Tables S2, S3). In the case where more than 25% of a previously identified comparison area was overlapped by a more recently licensed intervention area (central and/or buffer zone), that comparison area was removed from the analysis. In the case where a comparison area was overlapped by an intervention area but at least 75% of it remained as one continuous area, the comparison area boundaries (central and/or buffer zone) were redrawn so that its central zone was at least two km from the central zone of the intervention area. In the case of overlapping comparison areas central zones, herds were randomly allocated between the comparison areas and confounder data associated with the herds in each area recalculated. In the case of a comparison area buffer overlapping a comparison area central zone, the herds remained in the central zone and the boundary to the buffer was redrawn. In the case where buffer zones to different comparison areas overlapped, herds were randomly allocated between the buffers and confounder data associated with herds recalculated.

### Statistical analysis

The analytical approach was the same as that followed in Brunton *et al*.^11^. Effects in central zones and buffer zones were estimated separately because previous research has suggested that different effects in cattle TB in the areas surrounding the culling areas compared to areas where culling is conducted^10,11^. Crude OTF-W and all TB incidence rate ratios (IRRs) comparing incidence rates between intervention and comparison areas were calculated for three 12-month periods prior to the baseline date for all areas; and 12-month periods after the baseline date until the end of the follow-up (four years for Gloucestershire and Somerset and two years for Dorset).

Multivariable Poisson regression was conducted to measure changes in TB incidence over four or two years in central and buffer zones whilst controlling for confounding factors. IRRs were estimated for all variables as the exponent of the estimated Poisson regression coefficient. Matching of comparison areas to either Gloucestershire or Somerset was achieved through the inclusion of an area variable. The mathematical description of the model is as follows:

$$log({\mu }_{i})={\beta }_{0}+\mathop{\sum }\limits_{j=1}^{j=p}{\beta }_{j}{x}_{j}$$

where *μ*_*i*_ = the expected number of OTF-W or TB incidents for the areas during the time period under analysis, with the observed number being Poisson distributed with mean *μ*_*i*_, *β*_0_ = the intercept. Coefficients *β*_1_ to *β*_*p*_ are multiplied by explanatory variables *x*_1_to *x*_*p*_ including an intervention effect variable matching each intervention area (Gloucestershire, Somerset and Dorset) to its comparison areas.

The natural logarithm was taken of explanatory variables that were counts such as herd years of risk, herd size and number of badgers removed as part of historical badger control operations. Where a value was zero, 0.5 was added to the count before taking the logarithm. Effects by culling area (Gloucestershire, Somerset or Dorset) were measured by including a single intervention status variable and variables representing the geographical area (coded as Gloucestershire, Somerset or Dorset) which included both the intervention areas and their matched comparison areas. Statistical interaction by area was tested in the final models.

Using the dataset compiled for the current follow-up, OTF-W incidence rates were initially estimated in models including the same explanatory factors as in Brunton *et al*.^11^. Models were then rebuilt to investigate whether a better fit might be achieved with different covariates. Initial models included all explanatory factors shown to differ between intervention areas in the descriptive analyses. Predictors not statistically significantly associated with OTF-W incidents in the initial model were then eliminated. The subsequent model included terms for area, herd years at risk, OTF-W incidence rate in the three years up the baseline date when culling started and predictors that had been statistically significantly associated with OTF-W incidents in the initial model. Other predictors were then re-inserted one at time in a forward selection procedure whilst model fit was assessed using deviance^19^ and Pearson statistics^20^ and Akaike’s Information Criterion^21^ (AIC). Herd size and OTF-W incidence rate over the three years prior to baseline when culling started were retained in all models. Residuals were examined in the best fitting models and model stability examined by removing comparison areas that had the largest leverage statistics. Separate models were built to estimate effects for the individual year’s one, two, three and four since culling began. All TB incidence rates (OTF-W plus OTF-S incidents) were estimated in models including the same explanatory variables used in the models for OTF-W incidence rates.

Sensitivity analyses included (1) rerunning models reported in Brunton *et al*.^11^ for two years follow-up in Gloucestershire and Somerset with the new HYR, badger density, flood zone 3 and RBCT proactive area data (2) examining effects in final models with the removal of one comparison area at a time and (3) comparing effects in herds in the central zones subdivided into outer zone herds located within two km of the boundary and inner zone herds located two or more km inside the boundary.

The statistical analyses were conducted using Stata release 14.1 (Stata Corp., Texas, USA). Standard errors on effect estimates were calculated controlling for any non-Poisson variation (greater or smaller variance in the counts of OTF-W incidents between areas than would be expected by chance) using a robust estimator of variance in Stata^22,23^. Probability values (p values) of less than five percent (p < 0.05) were interpreted as statistically significant.

## Results

### Overlap between comparison areas and intervention areas licensed in 2016

Four comparison areas (including central and buffer zones) were excluded from the analyses because more than 25% of their land was overlapped by land in one of the eight intervention areas licenced between 2015 and 2016 (Tables S2, S3). Gloucestershire and Somerset intervention areas lost one comparison area each and Dorset lost two. Additionally, the land area and number of herds in the central zones of two comparison areas for Gloucestershire and one for Dorset were reduced due to intervention area overlap (Table S2). Land area and the number of herds in 21 buffer zones were also reduced due to intervention area or comparison area central zone overlap (Table S3).

### Baseline characteristics in intervention and comparison areas

Dorset intervention area herds were larger and more likely to be dairy than Gloucestershire and Somerset herds (Tables 1, 2). The estimated badger density prior to industry-led culling was almost twice as high in the Dorset intervention area compared to the Somerset and Gloucestershire areas and fewer badgers had been removed in historical operations. More of the Gloucestershire intervention area land was classified as flood zone 3 than in Somerset or Dorset. Somerset comparison areas were on average located further from the intervention area than Gloucestershire and Dorset comparison areas.

**Table 1:** Distribution of area attributes used to rank and match comparison areas to intervention areas.

Area and zones	Area size km^2^	All TB incidents prior to the baseline date		OTF-W incidents prior to the baseline date		Number of herds	Herd size median	Distance to intervention area km	RBCT proactive %
		Over 36 months	Over 12 months	Over 36 months	Over 12 months				
Gloucestershire									
Intervention central	310.8	90	18	69	16	215	46.0	0.0	0.0
Comparison central (n = 9)	308.8	75.4	25.6	60.9	21.7	160.2	45.6	34.7	0.6
Intervention buffer	143.2	41	17	29	11	94	47.0	0.0	0.3
Comparison buffer (n = 9)	160.9	44.4	15.1	36.3	12.8	83.8	56.7	31.6	3.7
Somerset									
Intervention central	256.2	106	30	85	27	154	53.5	0.0	0.0
Comparison central (n = 9)	255.7	76.4	27.1	61.7	22.1	162.1	52.8	60.3	0.0
Intervention buffer	180.3	43	16	36	13	88	39.5	0.0	12.1
Comparison buffer (n = 9)	133.2	38.2	13.4	29.6	10.9	84.3	60.3	60.3	2.0
Dorset									
Intervention central	223.3	80	26	57	17	157	100.0	0.0	0.0
Comparison central (n = 8)	217.0	59.1	19.4	44.5	15.9	129.4	91.3	44.5	0.0
Intervention buffer	172.2	37	10	23	8	113	95.0	0.0	0.0
Comparison buffer (n = 8)	109.3	31.9	13.9	22.1	10.0	75.4	77.1	44.3	0.0

Only OTF-W (Officially Tuberculosis Free Withdrawn, incidents of bovine tuberculosis (TB) in cattle herds with confirmatory evidence of *Mycobacterium bovis* infection from post-mortem tests) were used in matching of comparison areas. All TB incidents is OTF-W + OTF-S (OTF Suspended, which are TB incidents without confirmatory evidence of *M. bovis* infection from post-mortem tests). Comparison area values are means for 9, 9 and 8 areas for Somerset, Gloucestershire and Dorset respectively. Comparison areas for Somerset and Gloucestershire are the same as those reported in Brunton et al.^11^ but reduced by one for each area due to overlap of new interventions licensed in 2015 and 2016. Two Dorset comparison areas were lost due to overlapping intervention areas licensed in 2016. Values for each area are reported in the Supplement.

**Table 2:** Distribution of potential confounding factors across intervention and matched comparison areas.

Area and zones	Dairy herds %	Badger density/km^2^	Number of badgers removed historically			Flood zone category 3 %	Motorway total length/km	Urban area %	Number of farms	>1 land parcel in area %	All land inside area %	All land inside central or buffer zones %
			1972–1989	1990–1998	1999–2006							
Gloucestershire												
Intervention central	17.7	5.1	52	366	62	16.2	0	0	215	83.3	66.0	71.6
Comparison central (n = 9)	15.9	5.5	194.1	94.7	25.1	7.0	4.6	5.6	158.4	76.5	51.6	67.5
Intervention buffer	22.3	4.9	21	43	49	16.6	25.9	11.5	92	62.8	22.3	37.2
Comparison buffer (n = 9)	15.7	5.4	97.9	66.4	62.8	8.8	5.9	5.3	82.9	74.9	26.9	49.0
Somerset												
Intervention central	8.4	5.0	0	375	230	2.9	0	0	153	81.2	63.6	81.2
Comparison central (n = 9)	18.9	5.4	171.3	105.8	3.6	7.0	3.9	3.1	160.9	77.9	54.8	68.0
Intervention buffer	3.4	5.1	0	87	102	3.4	0	0	87	73.9	36.4	53.4
Comparison buffer (n = 9)	21.2	5.5	35.1	53.4	18.2	8.1	1.4	4.4	83.7	75.0	25.9	47.7
Dorset												
Intervention central	50.3	9.7	5	0	0	9.0	0	0.9	156	79.0	61.8	72.0
Comparison central (n = 8)	33.9	7.1	71.3	65.3	4.5	12.5	6.1	2.1	127.8	79.0	51.1	64.3
Intervention buffer	36.3	9.8	0	8	0	6.4	0	5.4	112	68.1	23.0	41.6
Comparison buffer (n = 8)	26.9	7.3	41.3	43.1	2.6	9.1	1.3	4.7	74.5	67.3	18.6	36.3

Comparison area values are means for 9, 9 and 8 areas for Somerset, Gloucestershire and Dorset respectively. Comparison areas for Somerset and Gloucestershire are the same as those reported in Brunton et al.^11^ but reduced by one for each area due to overlap of the intervention area in Dorset licensed in 2015 and seven new intervention areas licensed in 2016. The original badger density variable reported in Brunton et al.^11^, has been replaced with new estimation of badger density that takes into account survey data made available after the previous analysis. Values for each area are reported in the Supplement. Dairy herds is the percentage of cattle herds that are dairy herds according to the APHA cattle surveillance database.

Comparison areas were fairly similar to their matched intervention areas (Tables 1, 2). However, a higher proportion of herds were dairy in Dorset and there was almost twice as much land in flood zone 3 in Gloucestershire compared to their respective comparison area central zone averages. A lower proportion of Gloucestershire intervention area central zone farms had land parcels outside the area compared to its comparison central zone average. There was also twice as much motorway in the Gloucestershire intervention area buffer zone compared to its comparison area buffer zone average.

### Crude OTF-W incidence rates in intervention and comparison areas

In the four years since the baseline date when culling started there were 64 and 80 OTF-W incidents in the Gloucestershire and Somerset intervention central zones respectively. In the two years since the baseline date there were 44 OTF-W incidents in the Dorset intervention central zone. There were 34 and 40 OTF-W incidents respectively during four years in the Gloucestershire and Somerset intervention buffer zones and 13 incidents during two years in the Dorset intervention buffer zone.

Incidence rates over one and three years before the baseline date in each intervention area were correlated, particularly OTF-W incidence rates in the Dorset central zone (Spearman correlation coefficient 0.867, Spearman p value = 0.003). Annual crude OTF-W incidence rates were lower in the Gloucestershire intervention central zone compared to comparison areas from two years prior to the baseline date to four years post baseline when culling started (Table 3); with the strongest relative decline in the intervention area central zone in the fourth year after culling started. Rates in the Somerset intervention central zone were higher than the mean for comparison zones until the second year after culling started. Other differences between OTF-W between intervention and comparison area central and buffer zones were not as strong. Rates in comparison buffers were lower than rates in the intervention area buffers throughout the follow-up period in Gloucestershire and Dorset. Differences in rates for all TB incidents showed a similar pattern to the OTF-W rates (Table S4).

**Table 3:** Crude OTF-W incidence rates and incidence rate ratios (IRR) for central and buffer zones of intervention areas compared to central and buffers zones of comparison areas, for each 12-month period prior to and post the baseline date when culling started in each area.

12-month reporting period	Intervention central	Comparison central	IRR	95% confidence interval		*p* value	Intervention buffer	Comparison buffer	IRR	95% confidence interval		*p* value
Gloucestershire												
Year 3 prior	0.18	0.15	1.19	0.78	1.76	0.386	0.15	0.17	0.84	0.39	1.60	0.617
Year 2 prior	0.13	0.18	0.76	0.47	1.17	0.206	0.12	0.19	0.65	0.27	1.32	0.231
Year 1 prior	0.10	0.19	0.54	0.31	0.91	0.012	0.16	0.22	0.70	0.34	1.31	0.263
Year 1 post	0.13	0.20	0.65	0.39	1.02	0.052	0.15	0.19	0.78	0.38	1.46	0.447
Year 2 post	0.13	0.17	0.73	0.43	1.18	0.190	0.19	0.21	0.88	0.44	1.59	0.691
Year 3 post	0.12	0.16	0.72	0.41	1.19	0.185	0.07	0.15	0.44	0.12	1.18	0.088
Year 4 post	0.06	0.17	0.32	0.14	0.65	0.0002	0.12	0.14	0.83	0.32	1.80	0.666
Somerset												
Year 3 prior	0.22	0.17	1.32	0.85	1.97	0.181	0.12	0.15	0.78	0.34	1.55	0.496
Year 2 prior	0.26	0.15	1.73	1.13	2.56	0.009	0.19	0.15	1.26	0.66	2.24	0.413
Year 1 prior	0.24	0.20	1.24	0.80	1.86	0.299	0.19	0.18	1.07	0.55	1.91	0.800
Year 1 post	0.24	0.19	1.25	0.80	1.89	0.286	0.23	0.20	1.14	0.60	2.00	0.625
Year 2 post	0.17	0.18	0.92	0.55	1.46	0.742	0.15	0.17	0.91	0.42	1.75	0.797
Year 3 post	0.18	0.20	0.92	0.56	1.45	0.735	0.16	0.18	0.87	0.36	1.79	0.741
Year 4 post	0.12	0.18	0.65	0.34	1.15	0.129	0.14	0.18	0.76	0.32	1.57	0.473
Dorset												
Year 3 prior	0.15	0.14	1.03	0.58	1.73	0.876	0.09	0.11	0.81	0.31	1.80	0.634
Year 2 prior	0.17	0.15	1.10	0.67	1.73	0.652	0.09	0.10	0.90	0.37	1.93	0.820
Year 1 prior	0.15	0.16	0.90	0.51	1.50	0.701	0.09	0.17	0.55	0.23	1.13	0.087
Year 1 post	0.18	0.15	1.21	0.72	1.94	0.411	0.07	0.14	0.50	0.18	1.14	0.083
Year 2 post	0.20	0.14	1.42	0.87	2.24	0.135	0.08	0.12	0.68	0.26	1.49	0.338

Crude rates for all TB incidents (OTF-W and OTF-S) are shown in Table S4 in the Supplement. OTF-W = Officially Tuberculosis Free Withdrawn (*Mycobacterium bovis* infection confirmed by post-mortem tests), OTF-S = Officially Tuberculosis Free Suspended. Incidence rates are incidents (OTF-W or all TB) per herd years at risk.

After four years follow-up, 19.1% of the Gloucestershire and 15.6% of the Somerset herds in the central zone were no longer located in their respective areas. The percentage of Dorset herds that had moved after two years follow-up was 3.8% (Table S5).

### Adjusted OTF-W incidence rates in central zones of intervention and comparison areas

The fit of models for OTF-W incidence rates with the confounding factors reported in Brunton *et al*.^11^ was good for effects in the central zones over four years since baseline (deviance goodness-of-fit (gof) p = 0.506, Pearson gof p = 0.511) but poor for effects over two years (deviance gof p = 0.001, Pearson gof p = 0.001).

The best fitting model for effects in the central zone over four years since baseline showed statistically significantly lower OTF-W incidence rates in cattle herds in both Gloucestershire and Somerset intervention central zones relative to comparison central zones (Table 4, model A). This model had an AIC of 149.492 compared to an AIC of 152.847 for a model fitted to the same dataset using the same confounding factors as in Brunton *et al*.^11^. The effect was strongest in Gloucestershire where the central estimate was 66% lower than in comparison areas compared to 37% lower in Somerset (p = 0.038 for interaction by area). Comparison area labelled WS03 had the greatest leverage but its removal had little effect on estimates (Table S6).

**Table 4:** Multivariable Poisson regression models of the association between OTF-W incidence rates and four years culling in Somerset and Gloucestershire.

	IRR	Robust SE	p value	95% Confidence interval	
**Model A Central zones of Somerset and Gloucestershire**					
Intervention effect in Somerset area	0.63	0.03	<0.001	0.58	0.69
Intervention effect in Gloucestershire area	0.34	0.02	<0.001	0.29	0.39
Area = Somerset	1.10	0.07	0.134	0.97	1.25
Log transformed herds years at risk for 4 years of culling	4.26	0.57	<0.001	3.28	5.54
Log transformed OTF-W incidence rate over 3 years prior	1.26	0.09	0.001	1.10	1.45
Log transformed median herd size	0.86	0.25	0.593	0.48	1.51
Percentage of herds that were dairy	1.01	<0.01	<0.001	1.01	1.02
Distance to intervention (km)	1.00	<0.01	<0.001	0.99	1.00
Log transformed number of badgers culled historically	1.04	0.01	<0.001	1.02	1.06
Percentage of farms with at least 1 land parcel in area	1.02	0.01	0.007	1.00	1.03
**Model B Buffer zones of Somerset and Gloucestershire**					
Intervention effect in Somerset area	0.97	0.09	0.767	0.80	1.18
Intervention effect in Gloucestershire area	0.64	0.03	<0.001	0.58	0.70
Area = Somerset	1.22	0.06	<0.001	1.11	1.35
Log transformed herds years at risk for 4 years of culling	3.28	0.50	<0.001	2.43	4.44
Log transformed OTF-W incidence rate over 3 years prior	1.17	0.13	0.149	0.94	1.45
Log transformed median herd size	1.39	0.19	0.014	1.07	1.82
Percentage of land classed as urban	1.04	0.01	<0.001	1.03	1.05
Log transformed number of badgers culled historically	1.04	0.02	0.057	1.00	1.07
Percentage of farms with all land inside area	1.02	0.01	0.031	1.00	1.03

OTF-W = Officially Tuberculosis Free status Withdrawn. IRR = Incidence Rate Ratio. Intervention is industry led culling. SE = standard error. Observations = 20 in both models. Deviance goodness of fit (gof) p = 0.628 and 0.212, Pearson gof p = 0.637 and 0.197 for models A and B respectively.

The best fitting model for effects in the central zones over two years since baseline included a variable with quintiles of the distribution of the total number of badgers culled historically (Table 5, model C). This model had an AIC of 197.112 compared to an AIC of 221.735 for a model fitted to the same dataset using the same confounding factors as in Brunton *et al*.^11^. OTF-W incidence rates for Gloucestershire and Somerset intervention central zones were statistically significantly lower than in comparison central zones. There was no difference between OTF-W incidence rates in the Dorset intervention central zone compared to comparison central zones (p = 0.001 for interaction by area). Comparison area labelled WS01 had the greatest leverage but its removal had little effect on estimates (Table S6).

**Table 5:** Multivariable Poisson regression models of the association between OTF-W incidence rates and two years culling in Somerset, Gloucestershire and Dorset.

	IRR	Robust SE	p value	95% Confidence interval	
**Model C Central zones of Somerset, Gloucestershire and Dorset**					
Intervention effect in Somerset area	0.79	0.06	0.004	0.67	0.93
Intervention effect in Gloucestershire area	0.84	0.05	0.005	0.74	0.95
Intervention effect in Dorset area	1.10	0.08	0.168	0.96	1.27
Area = Somerset	1.01	0.09	0.901	0.85	1.20
Area = Dorset	0.69	0.18	0.151	0.41	1.15
Log transformed herds years at risk for 2 years of culling	2.34	0.25	<0.001	1.89	2.90
Log transformed OTF-W incidence rate over 3 years prior	1.59	0.11	<0.001	1.40	1.81
Log transformed median herd size	1.49	0.51	0.249	0.76	2.92
Percentage of land in flood zone 3	0.98	<0.01	<0.001	0.97	0.98
Distance to intervention (km)	1.00	<0.01	0.017	1.00	1.00
Between 0 and 1 badgers removed 1972–2006	Badgers removed reference category				
Between 2 and 35 badgers removed 1972–2006	0.99	0.10	0.891	0.81	1.20
Between 38 and 72 badgers removed 1972–2006	0.89	0.08	0.172	0.75	1.05
Between 79 and 356 badgers removed 1972–2006	1.41	0.15	0.001	1.15	1.73
Between 371 and 1589 badgers removed 1972–2006	0.91	0.07	0.210	0.77	1.06
**Model D Buffer zones of Somerset, Gloucestershire and Dorset**					
Intervention effect in Somerset area	1.09	0.10	0.360	0.91	1.30
Intervention effect in Gloucestershire area	0.89	0.14	0.446	0.66	1.20
Intervention effect Dorset	0.45	0.04	<0.001	0.37	0.54
Area = Somerset	1.09	0.07	0.175	0.96	1.24
Area = Dorset	0.80	0.09	0.035	0.65	0.98
Log transformed herds years at risk for 2 years of culling	2.45	0.27	<0.001	1.97	3.05
Log transformed OTF-W incidence rate over 3 years prior	1.33	0.04	<0.001	1.25	1.42
Log transformed median herd size	1.32	0.14	0.009	1.07	1.62
Percentage of land classed as urban	1.05	0.01	<0.001	1.04	1.07
Length of motorway (km)	1.00	<0.01	0.062	1.00	1.00
Distance to intervention (km)	1.00	<0.01	0.013	0.99	1.00
Percent. of farms with all land inside central/buffer zones	1.01	<0.01	0.001	1.01	1.02

OTF-W = Officially Tuberculosis Free status Withdrawn. IRR = Incidence Rate Ratio. Intervention is industry led culling. SE = Standard error. Observations = 29 in both models. Deviance goodness of fit (gof) p values were 0.529 and 0.707 and Pearson gof p values were 0.562 and 0.600 respectively for models C and D.

**Figure 2:**
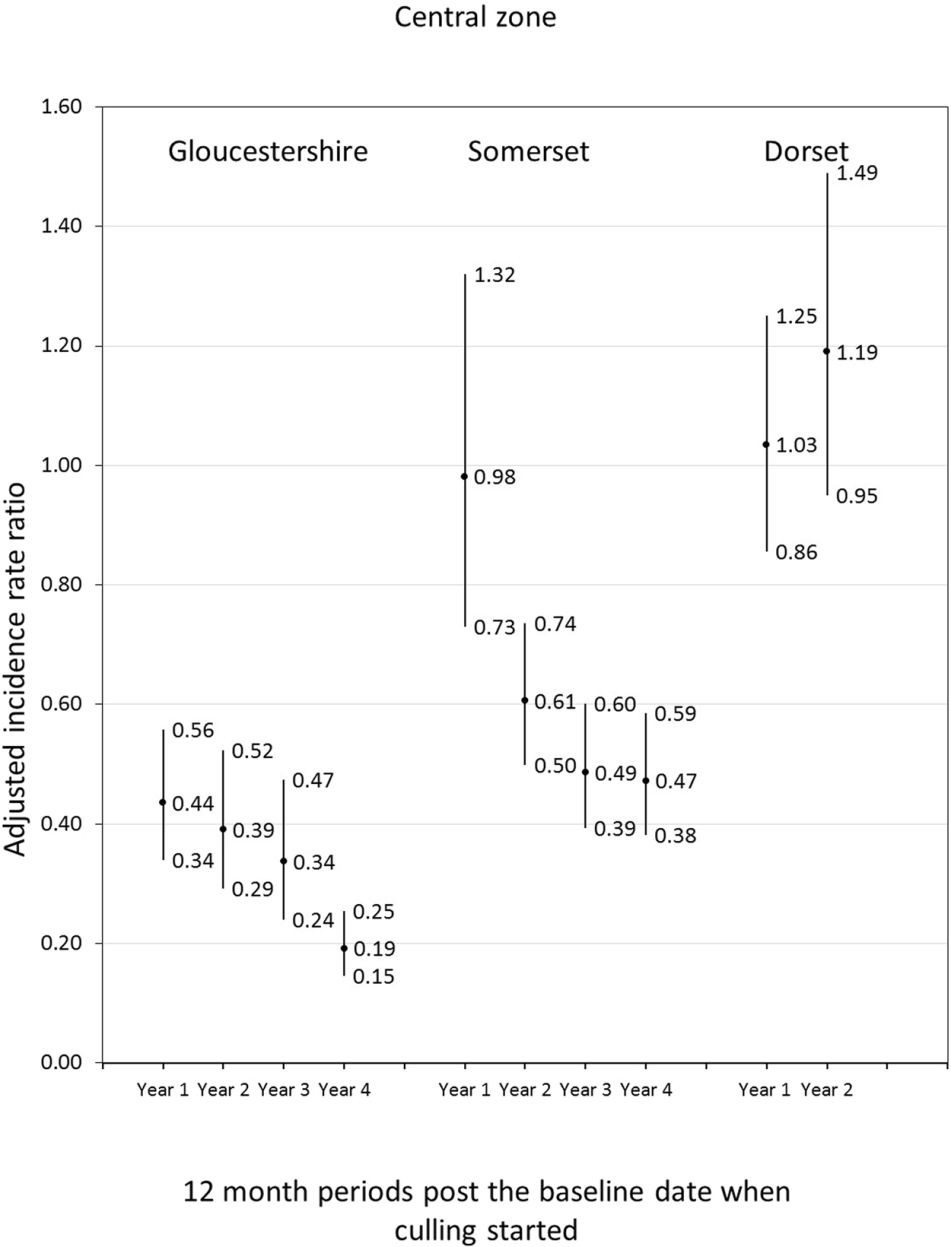
Adjusted incidence rate ratios (central estimates and 95% confidence intervals) of Officially Tuberculosis Free Withdrawn (OTF-W) incidence in cattle herds in the central zones of intervention areas compared to herds in the central zones of comparison areas. Annual incidence rate ratios (IRRs) for Gloucestershire and Somerset compared to comparison areas were estimated in Poisson regression models adjusting for the OTF-W incidence rate in the three years prior to baseline, median herd size, percentage of herds that were dairy, distance between intervention areas and comparison areas, the log transformed total number of badgers removed between 1972 and 2006 and the percentage of farms with at least one land parcel in the central zone. The annual IRRs for Dorset compared to comparison areas were estimated in models adjusting for effects in Somerset and Gloucestershire, the OTF-W incidence rate in the three years prior to baseline, median herd size, percentage of land in flood zone 3, distance between intervention areas and comparison areas and quintiles for total numbers of badgers removed between 1972 and 2006.

The central estimates for annual IRRs declined in Somerset and Gloucestershire with years since culling started (Fig. 2). The OTF-W incidence rates for the Gloucestershire intervention were lower than in comparison central zones each of the four years since baseline (p < 0.001). Rates for the Somerset intervention were no different to comparison rates in the first year since baseline but were statistically significantly lower in subsequent years (p < 0.001). Differences between annual rates in the Dorset intervention central zone and comparison central zones were not statistically significant.

### Adjusted OTF-W incidence rates in buffer zones of intervention and comparison areas

The fit of models for OTF-W incidence rates with the confounding factors reported in Brunton *et al*. ^11^ was not good for effects in the buffer zones over four years since culling started (deviance gof p = 0.035, Pearson gof p = 0.037) but good for effects over two years (deviance gof p = 0.443, Pearson gof p = 0.394).

The best fitting model for effects in the buffer zones over four years since baseline showed that rates were around 36% lower in the Gloucestershire intervention buffer zone relative to comparison buffer zones (p < 0.001, Table 4, model B). This model had an AIC of 139.210 compared to an AIC of 147.634 for a model fitted to the same dataset using the same confounding factors as in Brunton *et al*.^11^.There was no difference between incidence rates in the Somerset intervention and comparison buffer zones but also no evidence for interaction by intervention area (p = 0.396).

The best fitting model for effects in the buffer zones over two years showed that Dorset rates were around 55% lower than in comparison zones (p < 0.001, Table 5, model D). This model had an AIC of 170.884 compared to an AIC of 176.801 for a model fitted to the same dataset using the same confounding factors as in Brunton *et al*.^11^. The incidence rate was lower for the Gloucestershire buffer compared to comparison buffers but the difference was not statistically significant. There was no statistically significant difference in the rates in the Somerset buffer zone relative to comparison buffer zones. The interaction p value was highly statistically significant showing effects differed by intervention area (p < 0.001). Comparison area labelled WS03B had the greatest leverage in both the two year and the four year model for buffer zones but its removal did not change the interpretation of effects (Table S7).

**Figure 3:**
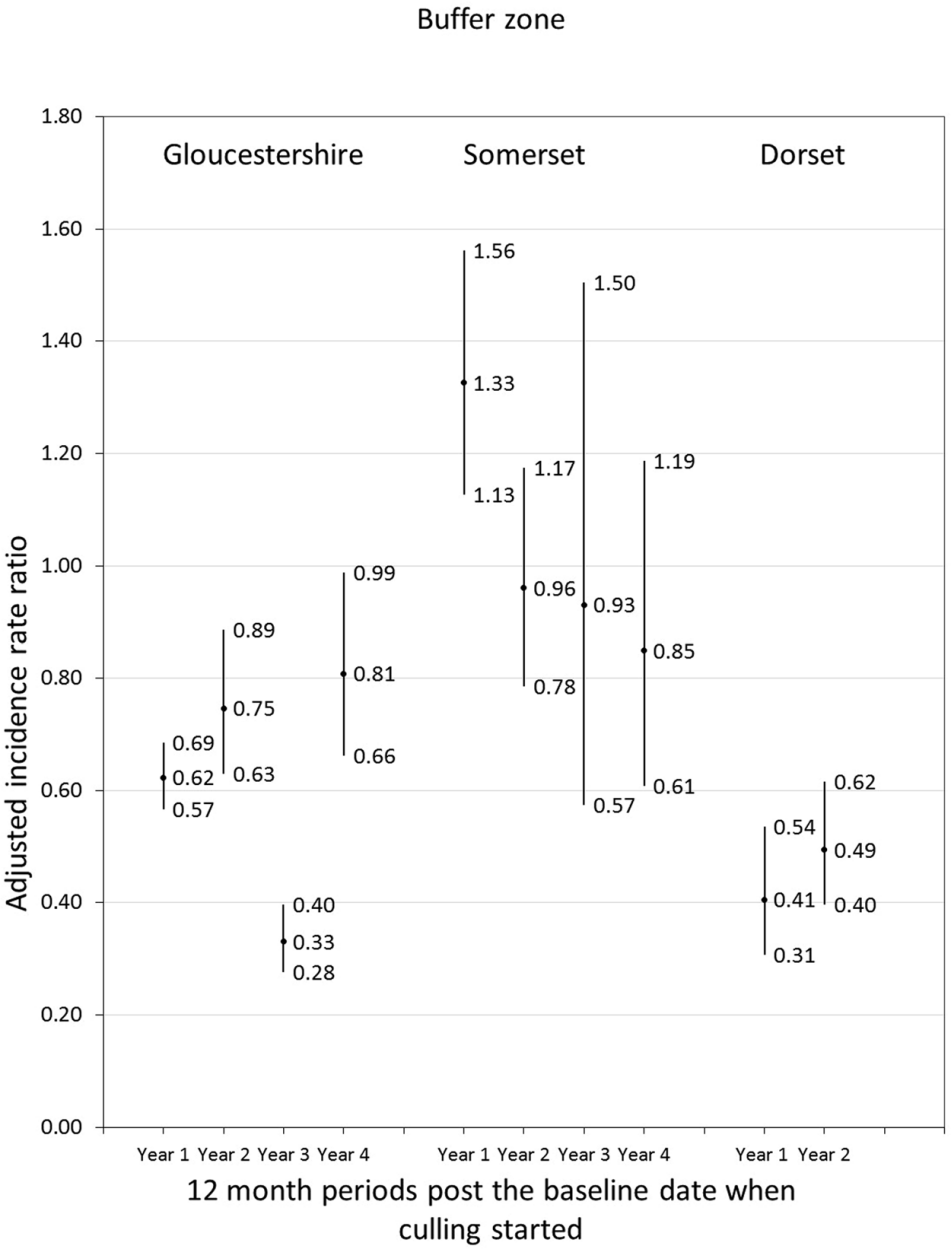
Adjusted incidence rate ratios (central estimates and 95% confidence intervals) for Officially Tuberculosis Free-Withdrawn (OTF-W) incidence in cattle herds in the buffer zones of intervention areas compared to herds in the buffer zones of comparison areas. Annual incidence rate ratios (IRRs) for Gloucestershire and Somerset compared to comparison areas were estimated in Poisson regression models adjusting for the OTF-W incidence rate in the three years prior to baseline, median herd size, percentage of urban land, the log transformed total number of badgers removed between 1972 and 2006 and the percentage of farms with all land in the buffer zone. Annual IRRs for Dorset compared to comparison areas were estimated in models adjusting for effects in Somerset and Gloucestershire, the OTF-W incidence rate in the three years prior to baseline, median herd size, percentage of urban land, length of motorway, distance between intervention areas and comparison areas and the percentage of farms with all land in the central or buffer zone.

Incidence rates in the Gloucestershire and Dorset intervention buffer zones were lower than in comparison buffer zones each year since culling started (Fig. 3). Incidence rates in the Somerset intervention buffer zone were higher than in comparison areas (p = 0.001) in the first year after culling started but were similar to comparison zones in years two, three and four.

### Adjusted all TB (OTF-W plus OTF-S) incidence rates in central and buffer zones

The adjusted IRRs for all TB incidents in the Somerset and Gloucestershire intervention central zones over four years were statistically significantly lower than in comparison areas zones (Table S8) but not over two years (Table S9). The Dorset intervention central zone had a statistically significantly higher incidence rate over two years than comparison areas zones (Table S9). Rates in Gloucestershire intervention buffer zone were statistically significantly lower than in comparison areas over four years, but not over two years. There were no differences between rates in the Somerset and Dorset intervention buffer zones to comparison area buffer zones.

### Sensitivity analyses

IRR estimates for the effects over two years in Somerset and Gloucestershire using the updated estimates for HYR, percentage of land exposed to RBCT proactive culling and badger density as opposed to badger sett density were very similar to those previously reported for the central zone in Brunton *et al*.^11^ (Table S10). However, the increase in incidence in the Somerset buffer zone was smaller and no longer statistically significant.

Removal of comparison areas from the final four and two year central and buffer zone models (Table 4, model A and Table 5, model C) had little effect on IRR estimates (Tables S6, S7).

**Figure 4:**
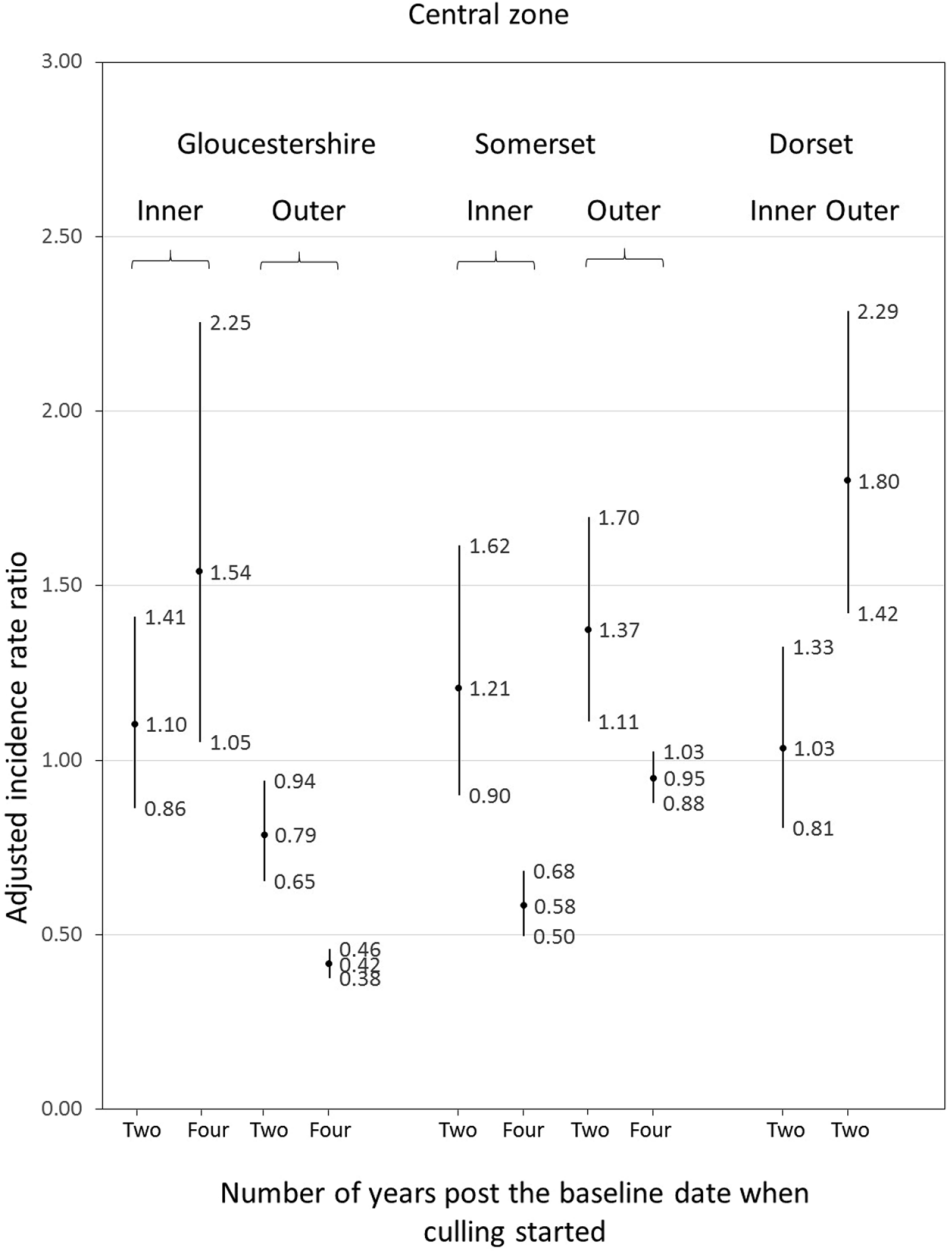
Adjusted incidence rate ratios (central estimates and 95% confidence intervals) for Officially Tuberculosis Free-Withdrawn (OTF-W) incidence in cattle herds in inner and outer zones of intervention central zones compared to in the inner and outer zones of the comparison area central zones. Inner zone herds were from farms with a geolocation two or more km inside the boundary to the central zone. Outer zone herds were from farms with a geolocation within two km of the boundary to the central zones. Annual incidence rate ratios (IRRs) for inner zone herds over four years were estimated in models adjusting for intervention area, OTF-W incidence rate in the three years prior to baseline, median herd size, percentage of flood zone 3 land and quintiles for total numbers of badgers removed between 1972 and 2006. Annual incidence rate ratios (IRRs) for inner zone herds over two years were estimated in models adjusting for intervention area, OTF-W incidence rate in the three years prior to baseline, median herd size, percentage of flood zone 3 land, percentage of dairy herds, badger density and the percentage of farms with all land in the central zone. Annual incidence rate ratios (IRRs) for outer zone herds over two years were estimated in models adjusting for intervention area, OTF-W incidence rate in the three years prior to baseline, median herd size, percentage of urban land, percentage of farms with at least one land parcel in area and percentage of land that was in a Randomised Badger Culling Trial (RBCT) Proactive area. Annual incidence rate ratios (IRRs) for outer zone herds over four years were estimated in models adjusting for intervention area, OTF-W incidence rate in the three years prior to baseline, median herd size, percentage of dairy herds, percentage of farms with at least one land parcel in area and percentage of land that was in a RBCT proactive area.

The Somerset intervention central zone had a higher proportion of herds (68.2%) located within two km of the boundary to the zone than Gloucestershire (53.5%) or Dorset (51.6%). There was no obvious pattern to differences in OTF-W incidence rates between inner and outer zones to the central zones (Fig. 4).

The central estimates for IRRs were the same but confidence intervals were generally wider in models without control for non-Poisson variation compared to models with control. The OTF-W incidence rates over four years in central zones were still statistically significantly lower in Gloucestershire and Somerset relative to comparison area zones as were OTF-W incidence rates in the Gloucestershire buffer zone (compare Table 4 to Table S11). Central estimates for OTF-W rates in Gloucestershire and Somerset central zones over two years were lower than comparison central zones but not statistically significantly so; there was no change to the interpretation of effects in Dorset (compare Table 5 to Table S12).

## Discussion

The results from this study showed that there were statistically significant decreases in cattle TB incidence in the Gloucestershire and Somerset intervention areas after four years of culling, consistent with an earlier analysis based on two years of badger culling^11^. The decreases in confirmed TB (OTF-W) incidence rates relative to comparison areas observed were 66% (95% CI 61 to 71%) and 37% (95% CI 31 to 42%) in Gloucestershire and Somerset respectively. However, there was no change in OTF-W incidence after two years of culling in Dorset central zone. Decreases in OTF-W incidence rates in the Gloucestershire and Dorset buffer zones relative to comparison area buffer zones were also observed, which were unexpected. Other research suggests that an increase in cattle TB may occur in areas surrounding culling areas due to increased *M. bovis* transmission caused by perturbation (increased mixing) of badger populations^24^.

Establishing causality between an intervention and a disease that involves transmission between two animal species is challenging. Each component of the causal pathway is affected by environmental factors that differ between areas such as the spatial distribution of badger habitat, the location of cattle and the effectiveness of the culling operations. Furthermore, evaluating control policies that change over time in response to new information and political climate adds additional challenges.

Since two original pilot culls of 2013, a further 29 licences for culling in other areas of the HRA have been issued. We included 10 comparison areas per intervention area in the study design because we had no control over the selection of future culling areas and anticipated comparison area land would be lost to newly licenced areas over time^18^. Four comparison areas have been lost and the land area of 21 buffer zones reduced because of overlap by culling areas licensed after the culling areas in Gloucestershire, Somerset and Dorset. Land that became part of a new culling area had to be excluded entirely from our analysis because herds on that land became exposed to culling. Incidence rates throughout the follow-up period were only calculated from the herds in existence on comparison central and buffer zone land when culling started (the baseline date) that also did not become licensed for culling at any point in the follow-up period. This was to ensure that the TB incidence rates for comparison zones were from herds that were not directly exposed to culling at any point. The total number of TB incidents recorded on comparison area land will be lower than if none of the land had been lost to culling. However the incidence rates (incidents per herd years at risk) should be an accurate reflection of disease rates in herds on comparison area land.

The RBCT was a rigorously conducted scientific trial and the best evidence of what might be achievable from badger removal on a large scale in England^9^. As a randomised controlled trial, it is less vulnerable to confounding by differences in the distribution of TB risk factors between areas, than the current study. We attempted to control for effects from confounding by adjusting for factors known to be associated with TB risk. However, these factors were derived from routinely collected surveillance data and may be subject to misclassification biases. There may have been factors associated with the granting of culling licenses e.g. greater uptake of biosecurity that could affect TB risk. We could not adjust for unknown confounders nor factors where we did not have information from both intervention and comparison areas. A reduction of 23% (95% CI 12 to 33%) in confirmed TB incidents in areas subject to at least four years of widespread systematic culling compared to non-culled areas was observed in the RBCT^6,10^. Reductions over four years in Gloucestershire and Somerset were larger. However, this increase may be because industry-led culling was conducted over a longer period each year and the areas culled were larger than in the RBCT.

Some heterogeneity in effects is to be expected due to chance and other factors e.g. differences in cattle distribution, the burden of infection in badger populations and culling coverage. There were some inconsistencies between our results and those of the RBCT as well as between areas. We did not find evidence for a stronger beneficial effect on cattle TB from culling with increasing distance from the boundary of the central zones. There was initially evidence for a larger beneficial effect with increasing distance inside culling areas in the RBCT, although the trend was non-statistically significant in later analyses^10,25^. Buffer zone incidence rates were statistically significantly lower in the Gloucestershire and Dorset buffer zones compared to comparison buffer zones throughout the follow-up period but statistically significantly higher in the Somerset buffer during the first year of the follow-up. In the RBCT, a 25% increase (95% CI -1% to 56%) in incidents was initially observed in the buffer zones around culled areas, which reduced over time^10^.

One third of buffer zones to Gloucestershire, Somerset and Dorset culling areas were reduced in size to take account of culling areas licensed later. It is likely that both cull coverage of areas and boundary permeability will have affected badger ranging behaviour. Up to 20% of intervention area cohort herds were no longer in existence in the area after four years follow-up and other herds had moved into the areas. However, trends in crude incidence rates in the cohort herds are similar to those for herds in existence in the areas on the baseline date and anniversaries of the baseline date^26^.

There has been debate about the delay that might be expected between culling badgers and an observable effect on the incidence of TB in cattle TB^27^. Behavioural data show that local reductions in badger density can cause badgers to alter their ranging behaviour within a few weeks^24^. Cattle most likely acquire infection from badgers from indirect contact, e.g. from pasture or contaminated feed^28^. Experimental work suggests pulmonary exposure of cattle to very low numbers of colony forming units of *M. bovis* will result in a positive SICCT test response within 12 weeks^29^. However detection is also dependent on the frequency of TB surveillance tests in the cattle herds (annual in the HRA during the follow-up period). There was no decline in TB incidence in cattle, between the first and second year of follow-up in Gloucestershire and Dorset central zones although there was in Somerset. This may reflect area differences in reduction in infection transmission and detection of infection. Furthermore, infection prevalence in the wildlife reservoir will vary between areas, affecting the relative impact of the badger removal policy.

As more areas are licensed for culling, they are likely to be less similar to RBCT areas and consequently the RBCT results may be less predictive. In contrast to Gloucestershire and Somerset, none of the Dorset area was within the RBCT. Dorset also contained larger herds, more dairy herds and had a higher estimated baseline density of badgers. The most recent badger population estimates imply that the proportion of the Dorset badger population culled in the first year at least, may have been lower than in Somerset and Gloucestershire (Table S13). For these reasons a reduction in the incidence of TB in cattle may take longer to emerge in Dorset. A longer follow-up increases power to detect effects^12^ and supports delaying analyses to a time point where there is strong evidence for sufficient power for robust evaluation of effects. The decline in cattle TB for the Gloucestershire and Somerset central zones over four years was slightly stronger and more robust than after two years of follow-up.

Martin *et al*.^30^ concluded that infected badgers explained 9–19% of cattle TB incidents in the East Offaly trial in Ireland. A more recent analysis of RBCT data estimated that 5.7% (95% CI 0.9–25%) of transmission to cattle herds is from badgers^31^. This alongside the level of reduction in OTF-W incidence rates observed in the current analysis suggests that there are other mechanisms at play that amplify effects associated with badger controls. Implementing culling may lead to greater focus on cattle controls, TB testing quality and implementation of biosecurity.

The results presented here, which were reasonably consistent with the RBCT, show that a culling policy implemented by the farming industry can result in statistically significant reductions in the incidence of cattle TB. However, given the observational nature of the study we cannot exclude entirely biases in our results due to for example, unknown or unmeasured confounding. We recommend that evaluation of the effects from culling continues. We need to know whether the beneficial effects that have been observed on cattle TB continue and can also be observed in other culled areas. We also need to understand why an increase in TB incidence rates in cattle has not been detected in buffer zones surrounding culling areas. The analysis highlights the difficulties in predicting effects from large scale interventions aimed to reduce infection transmission between animal species. Culling badgers will not provide the entire solution to the cattle TB problem in Great Britain^32^ and the impact of the policy needs to be evaluated alongside other TB controls.

## Supplementary information

Supplementary Information

Dataset1

## Data Availability

The datasets generated in September 2018 and analysed for the current study are available within the Supplement.

## References

[1] Animal and Plant Health Agency (APHA). Surveillance data for 2017 and historical trends. https://www.gov.uk/government/publications/bovine-tb-epidemiology-and-surveillance-in-great-britain-2017 (2018).

[2] Department for Environment, Food & Rural Affairs (Defra). The Strategy for achieving Officially Bovine Tuberculosis Free status for England. https://assets.publishing.service.gov.uk/government/uploads/system/uploads/attachment_data/file/300447/pb14088-bovine-tb-strategy-140328.pdf (2014).

[3] Clifton-Hadley RS, Wilesmith JW, Richards MS, Upton P, Johnston S (1995). The occurrence of *Mycobacterium bovis* infection in cattle in and around an area subject to extensive badger (*Meles meles*) control. Epidemiol Infect..

[4] Eves JA (1999). Impact of badger removal on bovine tuberculosis in east County Offaly. Irish Vet J..

[5] Griffin JM (2005). The impact of badger removal on the control of tuberculosis in cattle herds in Ireland. Prev Vet Med..

[6] Donnelly CA (2006). Positive and negative effects of widespread badger culling on tuberculosis in cattle. Nature..

[7] Department for Environment, Food & Rural Affairs (Defra). Guidance to Natural England Licences to kill or take badgers for the purpose of preventing the spread of bovine TB under section 10(2)(a) of the Protection of Badgers Action 1992. https://assets.publishing.service.gov.uk/government/uploads/system/uploads/attachment_data/file/710537/tb-licensing-guidance-ne.pdf (2018).

[8] Department for Environment, Food & Rural Affairs (Defra). Guidance to Natural England Licences to kill or take badgers for the purpose of preventing the spread of bovine TB under section 10(2)(a) of the Protection of Badgers Act 1992 (2015).

[9] Bourne, J. F. *et al*. Bovine TB: The Scientific Evidence. A Science Base for a Sustainable Policy to Control TB in Cattle. An Epidemiological Investigation into Bovine Tuberculosis. Final Report of the Independent Scientific Group on Cattle TB presented to the Secretary of State for Environment, Food and Rural Affairs the Rt Hon David Miliband MP (2007).

[10] Donnelly CA (2007). Impacts of widespread badger culling on cattle tuberculosis: concluding analyses from a large-scale field trial. Int J Infect Dis..

[11] Brunton LA (2017). Assessing the effects of the first 2 years of industry-led badger culling in England on the incidence of bovine tuberculosis in cattle in 2013–2015. Ecol Evol..

[12] Donnelly CA, Bento AI, Goodchild AV, Downs SH (2015). Exploration of the power or routine surveillance data to assess the impacts of industry-led badger culling on bovine tuberculosis incidence in cattle herds. Vet Rec..

[13] Downs SH (2013). Tuberculin manufacturing source and breakdown incidence rate of bovine tuberculosis in British cattle, 2005–2009. Vet Rec..

[14] Department for Environment, Food & Rural Affairs (Defra). Background and methodology to the National Statistics on the incidence of tuberculosis (TB) in cattle in Great Britain. https://assets.publishing.service.gov.uk/government/uploads/system/uploads/attachment_data/file/726116/bovinetb-annex-18jul18.pdf (2018b).

[15] Brunton LA (2015). A novel approach to mapping and calculating the rate of spread of endemic bovine tuberculosis in England and Wales. Spat Spatiotemporal. Epidemiol..

[16] Judge J, Wilson GJ, Macarthur R, Delahay RJ, McDonald RA (2014). Density and abundance of badger social groups in England and Wales in 2011–2013. Sci Rep..

[17] Judge J, Wilson GJ, Macarthur R, McDonald RA, Delahay RJ (2017). Abundance of badgers (*Meles meles*) in England and Wales. Sci Rep..

[18] Animal Health and Veterinary Laboratories Agency (AHVLA). Developing a surveillance system to report TB in cattle herds exposed to badger control in England: Protocol for phase one. Date 18.6.14. (2014).

[19] Nelder JA, Wedderburn RWM (1972). Generalised linear models. Journal of the Royal Statistical Society. Series A (General)..

[20] Pearson Karl (1900). X. On the criterion that a given system of deviations from the probable in the case of a correlated system of variables is such that it can be reasonably supposed to have arisen from random sampling. The London, Edinburgh, and Dublin Philosophical Magazine and Journal of Science.

[21] Akaike H (1974). A new look at the statistical model identification. IEEE transactions on Automatic Control.

[22] Huber, P. J. The behavior of maximum likelihood estimates under nonstandard conditions. In Vol. 1 of *Proceedings of the Fifth Berkeley Symposium on Mathematical Statistics and Probability*, 221–233 (Berkeley: University of California Press, 1967).

[23] White H (1982). Maximum likelihood estimation of misspecified models. Econometrica..

[24] Woodroffe R (2006). Effects of culling on badger *Meles meles* spatial organisation: implications for the control of bovine tuberculosis. J Appl Ecol..

[25] Jenkins HE, Woodroffe R, Donnelly CA (2010). The duration of the effects of repeated widespread badger culling on cattle tuberculosis following the cessation of culling. PloS one.

[26] Animal and Plant Health Agency (APHA). Bovine TB in cattle in badger control areas: Incidence monitoring report for the period 2013–2017. https://assets.publishing.service.gov.uk/government/uploads/system/uploads/attachment_data/file/750986/bovine-tb-in-cattle-badger-control-areas-monitoring-report-2013-2017.pdf (2018b).

[27] More SJ (2007). Does reactive badger culling lead to an increase in tuberculosis in cattle?. Vet Rec..

[28] Woodroffe R (2016). Badgers prefer cattle pasture but avoid cattle: implications for bovine tuberculosis control. Ecol Lett..

[29] Dean GS (2005). Minimum infective dose of *Mycobacterium bovis* in cattle. Infect Immun..

[30] Martin SW (1997). The association between the bovine tuberculosis status of herds in the East Offaly Project Area, and the distance to badger setts, 1988-1993. Prev Vet Med..

[31] Donnelly, C.A., Nouvellet, P. The Contribution of Badgers to Confirmed Tuberculosis in Cattle in High-Incidence Areas in England. *PLOS Currents Outbreaks. Edition* **1**, 10.1371/currents.outbreaks.097a904d3f3619db2fe78d24bc776098 (2013). 10.1371/currents.outbreaks.097a904d3f3619db2fe78d24bc776098 PMC3992815 24761309

[32] Godfray, H.C., Donnelly, C.A., Hewinson, G., Winter, M., Wood, J. Bovine TB strategy review. https://assets.publishing.service.gov.uk/government/uploads/system/uploads/attachment_data/file/755515/bovine-tb-review-2018.pdf (2018)

