# Supplementary material for: Assessing effects from four years of industry-led badger culling in England on the incidence of bovine tuberculosis in cattle, 2013–2017
Source: Sci Rep. 2019 Oct 11;9:14666. doi: 10.1038/s41598-019-49957-6 (PMC6789095; doi:10.1038/s41598-019-49957-6)
Supplement: Supplementary file 1 — Supplementary Information [file 41598_2019_49957_MOESM1_ESM.docx]

**Supplement**

**Assessing effects from four years of industry-led badger culling in England on the incidence of bovine tuberculosis in cattle, 2013-2017.**

Sara H. Downs, Alison Prosser, Adam Ashton, Stuart Ashfield, Lucy A. Brunton, Adam Brouwer, Paul Upton, Andrew Robertson, Christl A. Donnelly, Jessica E. Parry.

**Table S1.** Baseline dates and follow-up periods.

| Intervention area | Cull year | Start date | End date |
| --- | --- | --- | --- |
| Gloucestershire |  |  |  |
|  | Year 3 prior | 03/09/2010 | 02/09/2011 |
|  | Year 2 prior | 03/09/2011 | 02/09/2012 |
|  | Year 1 prior | 03/09/2012 | 02/09/2013 |
|  | Year 1 post | 03/09/2013* | 02/09/2014 |
|  | Year 2 post | 03/09/2014 | 02/09/2015 |
|  | Year 3 post | 03/09/2015 | 02/09/2016 |
|  | Year 4 post | 03/09/2016 | 02/09/2017 |
| Somerset |  |  |  |
|  | Year 3 prior | 26/08/2010 | 25/08/2011 |
|  | Year 2 prior | 26/08/2011 | 25/08/2012 |
|  | Year 1 prior | 26/08/2012 | 25/08/2013 |
|  | Year 1 post | 26/08/2013* | 25/08/2014 |
|  | Year 2 post | 26/08/2014 | 25/08/2015 |
|  | Year 3 post | 26/08/2015 | 25/08/2016 |
|  | Year 4 post | 26/08/2016 | 25/08/2017 |
| Dorset |  |  |  |
|  | Year 3 prior | 31/08/2012 | 30/08/2013 |
|  | Year 2 prior | 31/08/2013 | 30/08/2014 |
|  | Year 1 prior | 31/08/2014 | 30/08/2015 |
|  | Year 1 post | 31/08/2015* | 30/08/2016 |
|  | Year 2 post | 31/08/2016 | 30/08/2017 |

*Baseline date when culling started. Culling was conducted for minimum of six-weeks from the baseline date and then annually for six-week periods on the anniversary of the baseline date.

**Description of the method to estimate badger density**

Estimates of badger density were produced for all intervention areas, comparison areas and 2km buffers. The principal aim of badger density estimates was to produce a relative measure of abundance that could be used to compare (and control for) potential differences among areas in subsequent analyses.

Badger density estimates were produced using data from the national badger survey of England and Wales carried out in 2011-2013, which involved the surveying of >1600 randomly located 1km survey squares for badger field signs (see Judge *et al*. 2014 for details). Subsequent genotyping of hairs collected from a subset of social groups (located in these squares) was also used to produce estimates of badger social group size and badger density (see Judge *et al*. 2017 for details).

These studies both demonstrate that badger main sett (large burrow systems indicative of badger social groups) density and social group size vary among broad habitat categories, with larger groups and higher densities observed in pastoral habitats then upland or arable habitats (Judge *et al*. 2014, 2017).

To produce localised intervention area (or comparison area) level estimates of badger density we subsampled from the national survey data using a step wise process with the following stages.

1. **Calculate the land class composition of the area.** As badger density varies with land class type, the area of each land class is calculated for the area of interest. Land class data and definitions were taken from on the Institute of Terrestrial Ecology (now Centre for Ecology and Hydrology) Land Classification System (Bunce *et al*. 1981) as used in Judge et al. (2014). This data divides the UK into broad land class categories numbered from LC1 to LC7. *For example an area may be 200 km^2^ consisting of 150km^2^ of LC4 and 50km^2^ of LC1.*
2. **Subset the national survey data.** A 20km buffer is placed around the area of interest and all 1km national survey squares within this area are selected. Essentially this selects the ‘local’ badger activity data for that area.
3. **Estimate the number of badger social groups (main setts).** The subset of national survey data is randomly resampled (with replacement) a number of times to produce a sample with equal size to the intervention area. This is done for each habitat category and the number of main setts is summed to produce a total number of social groups. *For example if the area consists of* *150km^2^ of LC4 and 50km^2^ of LC1, the local data is resampled sampled to produce a 150km^2^ of LC4 and 50km^2^ of LC1, and the number of main setts is summed to produce an estimate of social groups in each area.*
4. **Calculate badger density.**  For each habitat type the social group size estimates (for that habitat type are randomly sampled (with replacement) to produce a number of social group sizes equal to the number of social groups in each area (calculated in step 3). The sum of these numbers for each habitat gives a total badger population for the area. This is then divided by the total area size to produce an estimate of badger density.
5. **Repeat the process to produce a mean badger density and measure of variance.** Steps 3 and 4 are repeated 1000 times, producing 1000 population estimates which are used to produce a mean badger density for that area

References

Bunce, R. G. H., Barr, C. J. & Whittaker, H. A. Land classes in Great Britain: preliminary descriptions for use of the Merlewood method of land classification. (Grange-over-Sands, Institute of Terrestrial Ecology, 1981).

Judge, J., Wilson, G.J., Macarthur, R., Delahay, R.J. & McDonald, R.A. Density and abundance of badger social groups in England and Wales in 2011–2013. Scientific Reports, 4 (2014).

Judge, J., Wilson, G.J., Macarthur, R., McDonald, R.A. & Delahay, R.J. Abundance of badgers (Meles meles) in England and Wales. Scientific Reports, 7 (2017).

**Overlap between areas; rules for area and herd allocation**

Each intervention area was allocated 10 comparison areas that most closely matched it in terms of TB risk that were not within a previously licenced intervention areas or within two km of an intervention area boundary. Cattle herds in existence in comparison area central and buffer zones on the baseline date when culling started in the matched intervention area, formed the cohort of herds compared to the cohort of herds in the intervention area central and buffer zones.

Rules were developed for the area and herd allocation as a result of overlapping by new intervention areas (Dorset in 2015 and seven others in 2016) and between comparison areas from Gloucestershire, Somerset and Dorset. The effects from the rules were as follows:

1. Each herd was only allocated to one area
2. Where a comparison area is overlapped by an intervention area:
   - Comparison area was removed from analysis if an intervention area (central zone plus buffer zone) overlapped > 25% of the comparison area (> 75% of any comparison area central zone must remain as a continuous land area)
   - Comparison area was reduced in size and herds allocated to intervention area where intervention areas overlapped <25% of comparison area land
3. Where two or more comparison area central zones overlap:
   - Herds were randomly allocated between areas
4. Where a comparison buffer zone is overlapped by a comparison area central zone
   - Herds were allocated to the central zone of the overlapping comparison area
5. Where a buffer zone overlaps another comparison area buffer zone:
   - Herds were randomly allocated between buffer zones


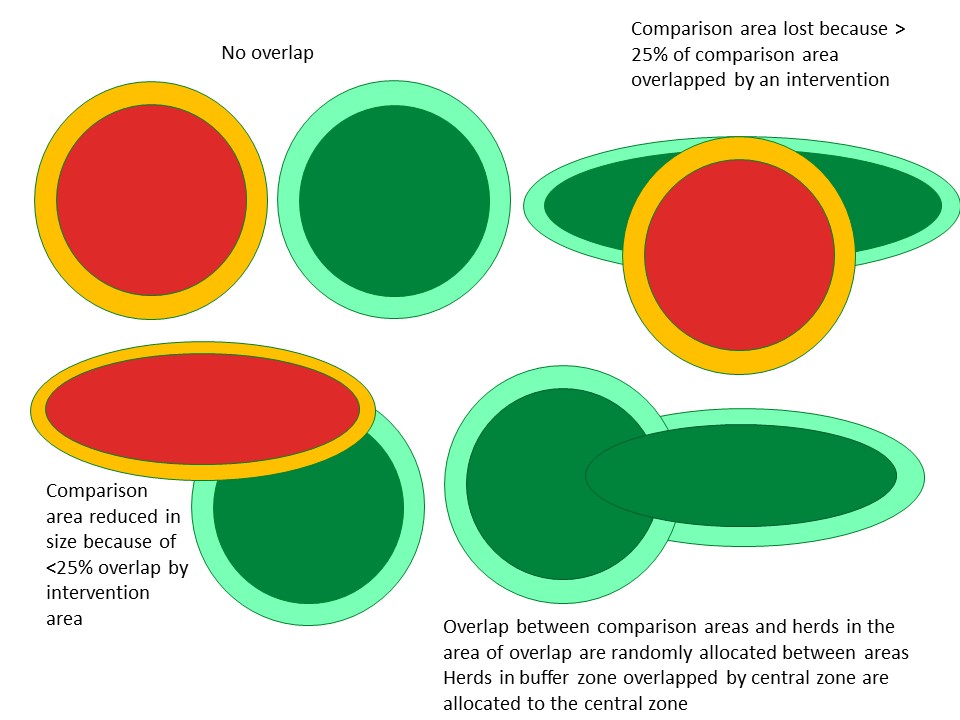


**Figure S1.** Area and herd allocation with overlap between areas

Footnote to Figure S1: Red and orange= intervention central and buffer zones; dark green and pale green =comparison area central and buffer zones

**Table S2.** Number of areas that overlap each intervention and comparison area central zone, percentage of original area size and herds retained for analysis

| Area label | Percentage of original area size remaining | Percentage of original herds remaining | New intervention central zones that overlap area | New intervention buffer zones that overlap area | Comparison central zones that overlap area | Comparison buffer zones that overlap area | Decision |
| --- | --- | --- | --- | --- | --- | --- | --- |
| **Gloucestershire** | |  |  |  |  |  |  |
| WG00^a^ | 100 | 100 | 1 | 2 |  |  | A |
| WG01 | 82.4 | 86.9 | 1 | 1 |  |  | B, D |
| WG02 | 99.7 | 99.4 |  | 1 |  |  | D, B |
| WG03 | 100 | 85.1 |  |  | 1 | 2 | B, E, F |
| WG04 | 100 | 82.6 |  |  | 2 | 3 | B, E, F |
| WG05 | 100 | 90.5 |  |  | 3 | 3 | B, E,F |
| WG06 | 100 | 100 |  |  |  |  | A |
| WG07 | 100 | 92.8 |  |  | 1 | 1 | B,E,F |
| WG08 | 100 | 71.3 |  |  | 2 | 2 | B,E,F |
| WG09 | 26.7 | 0 | 1 | 1 |  |  | C, D |
| WG10 | 100 | 100 |  |  |  |  | A |
| **Somerset** |  |  |  |  |  |  |  |
| WS00^a^ | 100 | 100 |  |  |  |  | A |
| WS01 | 100 | 100 |  |  |  |  | A |
| WS02 | 100 | 100 |  |  |  |  | A |
| WS03 | 100 | 97.1 |  |  | 1 | 1 | B,E,F |
| WS04 | 100 | 85.2 |  |  | 1 | 1 | B,E,F |
| WS05 | 100 | 92.8 |  |  | 1 | 1 | B,E,F |
| WS06 | 24.3 | 0 | 1 | 1 |  |  | C, D |
| WS07 | 100 | 56.9 |  |  | 3 | 3 | B,E,F |
| WS08 | 100 | 76.6 |  |  | 1 | 1 | B,E,F |
| WS09 | 100 | 91.9 |  |  | 1 | 1 | B,E,F |
| WS10 | 100 | 68.4 |  |  | 4 | 3 | B,E,F |
| **Dorset** |  |  |  |  |  |  |  |
| DR00^a^ | 100 | 100 |  |  |  |  | A |
| DR01 | 100 | 90.4 |  |  | 2 | 3 | B,E,F |
| DR02 | 100 | 79.8 |  |  | 2 | 2 | B,E,F |
| DR03 | 100 | 100 |  |  |  |  | A |
| DR04 | 100 | 59.5 |  |  | 3 | 3 | B,E,F |
| DR05 | 72.6 | 0 | 1 | 1 |  |  | C, D |
| DR06 | 79.2 | 69.9 | 1 | 1 | 1 | 1 | B,D, E,F |
| DR07 | 100 | 62.4 |  |  | 2 | 2 | B,E,F |
| DR08 | 100 | 100 |  |  |  |  | A |
| DR09 | 26.05 | 0 | 1 | 1 |  |  | C, D |
| DR10 | 100 | 86.7 |  |  | 1 | 2 | B,E,F |

Footnote: ^a^ Intervention central zone. A= No change, B= Number of herds in cohort reduced, C=Area omitted, D=Herds in comparison area allocated to intervention area (including cull areas licenced for 2016), E=Random allocation of herds between overlapping comparison area central zones, F=Herds in buffer zone assigned to comparison area central zone, G=Herds randomly allocated between buffer zones.

**Table S3.** Number of areas that overlap each intervention and comparison area buffer zone, percentage of original area size and herds retained for analysis.

| Area label | Percentage of original area size remaining | Percentage of original herds remaining | New  intervention central zones that overlap area | New intervention buffer zones that overlap area | Comparison central zones that overlap area | Comparison buffer zones that overlap area | Decision |
| --- | --- | --- | --- | --- | --- | --- | --- |
| **Gloucestershire** | |  |  |  |  |  |  |
| WG00B^a^ | 69.8 | 77.7 | 2 | 2 |  |  | B, D |
| WG01B | 80.7 | 95.4 | 1 | 1 |  |  | B, D |
| WG02B | 93.5 | 95.1 |  | 1 |  |  | D |
| WG03B | 74.2 | 69.9 |  |  | 2 | 2 | B, F, G |
| WG04B | 72.5 | 53.8 |  |  | 3 | 4 | B, F, G |
| WG05B | 71.7 | 55.2 |  |  | 4 | 3 | B, F, G |
| WG06B | 100 | 100 |  |  |  |  | A |
| WG07B | 90 | 77.7 |  |  | 1 | 1 | B, F, G |
| WG08B | 73.6 | 61.7 |  | 1 | 2 | 2 | B, D, F, G |
| WG09B | 0 | 0 | 1 | 1 |  |  | C, D |
| WG10B | 100 | 100 |  |  |  |  | A |
| **Somerset** |  |  |  |  |  |  |  |
| WS00B^a^ | 100 | 100 |  |  |  |  | A |
| WS01B | 100 | 100 |  |  |  |  | A |
| WS02B | 100 | 100 |  |  |  |  | A |
| WS03B | 90.6 | 90.7 |  |  | 1 | 1 | B, F, G |
| WS04B | 75.8 | 62.5 |  |  | 1 | 2 | B, F, G |
| WS05B | 79.2 | 85.4 |  |  | 1 | 1 | B, F, G |
| WS06B | 0 | 0 | 1 | 1 |  |  | C, D |
| WS07B | 29.8 | 18.9 |  | 1 | 3 | 3 | B, D, F, G |
| WS08B | 85.8 | 78.4 |  |  | 1 | 1 | B, F, G |
| WS09B | 73.7 | 62.2 |  |  | 1 | 1 | B, F, G |
| WS10B | 39.7 | 26.6 |  |  | 3 | 3 | B, F, G |
| **Dorset** |  |  |  |  |  |  |  |
| DR00B^a^ | 100 | 100 |  |  |  |  | A |
| DR01B | 57.2 | 55.2 |  |  | 3 | 3 | B, F, G |
| DR02B | 80 | 51.4 |  |  | 1 | 1 | B, F, G |
| DR03B | 100 | 100 |  |  |  |  |  |
| DR04B | 33.6 | 32.2 |  |  | 3 | 3 | B, F, G |
| DR05B | 0 | 0 | 1 | 1 |  |  | C, D |
| DR06B | 47.3 | 45.1 | 1 | 1 | 1 | 1 | B, D, F, G |
| DR07B | 35.8 | 31.8 |  |  | 2 | 2 | B, F, G |
| DR08B | 100 | 100 |  |  |  |  | A |
| DR09B | 0 | 0 | 1 | 1 |  |  | C, D |
| DR10B | 63.5 | 53.4 |  |  | 2 | 2 | B, F, G |

Footnote: ^a^ Intervention buffer zone. A= No change, B= Number of herds in cohort reduced, C=Area omitted, D=Herds in comparison area allocated to intervention area central zone or buffer (including cull areas licenced for 2016), E=Random allocation of herds between overlapping comparison area central zones, F=Herds in buffer zone assigned to comparison area central zone, G=Herds randomly allocated between buffer zones.

**Table S4.** Crude TB (OTF-W+OTF-S) incidence rates and incidence rate ratios (IRRs) for central and buffer zones of intervention areas compared to central and buffers zones of comparison areas, for each 12-month period prior to and post the baseline date when culling started in each area.

| 12-month reporting period | Intervention central | Comparison central | IRR | 95% confidence interval | | *p* value | Intervention buffer | Comparison buffer | IRR | 95% confidence interval | | *p* value |
| --- | --- | --- | --- | --- | --- | --- | --- | --- | --- | --- | --- | --- |
| Gloucestershire |  |  |  |  |  |  |  |  |  |  |  |  |
| Year 3 prior | 0.24 | 0.19 | 1.26 | 0.88 | 1.76 | 0.186 | 0.19 | 0.21 | 0.91 | 0.47 | 1.61 | 0.771 |
| Year 2 prior | 0.18 | 0.22 | 0.81 | 0.54 | 1.19 | 0.279 | 0.17 | 0.24 | 0.69 | 0.34 | 1.28 | 0.235 |
| Year 1 prior | 0.12 | 0.22 | 0.52 | 0.30 | 0.84 | 0.004 | 0.24 | 0.26 | 0.92 | 0.52 | 1.53 | 0.768 |
| Year 1 post | 0.18 | 0.24 | 0.77 | 0.51 | 1.14 | 0.186 | 0.20 | 0.22 | 0.90 | 0.49 | 1.54 | 0.716 |
| Year 2 post | 0.16 | 0.21 | 0.75 | 0.47 | 1.15 | 0.174 | 0.26 | 0.24 | 1.08 | 0.61 | 1.79 | 0.752 |
| Year 3 post | 0.16 | 0.22 | 0.72 | 0.45 | 1.11 | 0.128 | 0.15 | 0.21 | 0.71 | 0.32 | 1.40 | 0.327 |
| Year 4 post | 0.10 | 0.22 | 0.48 | 0.26 | 0.81 | 0.003 | 0.22 | 0.17 | 1.32 | 0.67 | 2.38 | 0.358 |
| Somerset |  |  |  |  |  |  |  |  |  |  |  |  |
| Year 3 prior | 0.27 | 0.21 | 1.27 | 0.86 | 1.82 | 0.205 | 0.16 | 0.21 | 0.76 | 0.38 | 1.38 | 0.371 |
| Year 2 prior | 0.37 | 0.19 | 1.97 | 1.38 | 2.76 | <0.001 | 0.20 | 0.19 | 1.05 | 0.57 | 1.82 | 0.825 |
| Year 1 prior | 0.27 | 0.24 | 1.12 | 0.74 | 1.64 | 0.541 | 0.24 | 0.22 | 1.06 | 0.59 | 1.80 | 0.793 |
| Year 1 post | 0.26 | 0.22 | 1.17 | 0.77 | 1.73 | 0.425 | 0.24 | 0.24 | 1.03 | 0.56 | 1.77 | 0.889 |
| Year 2 post | 0.19 | 0.21 | 0.91 | 0.56 | 1.40 | 0.686 | 0.18 | 0.21 | 0.86 | 0.43 | 1.57 | 0.652 |
| Year 3 post | 0.23 | 0.25 | 0.92 | 0.59 | 1.38 | 0.707 | 0.22 | 0.23 | 0.96 | 0.47 | 1.79 | 0.941 |
| Year 4 post | 0.15 | 0.25 | 0.60 | 0.33 | 0.99 | 0.035 | 0.20 | 0.24 | 0.86 | 0.43 | 1.57 | 0.648 |
| Dorset |  |  |  |  |  |  |  |  |  |  |  |  |
| Year 3 prior | 0.22 | 0.21 | 1.06 | 0.67 | 1.63 | 0.763 | 0.15 | 0.16 | 0.91 | 0.43 | 1.72 | 0.797 |
| Year 2 prior | 0.21 | 0.21 | 1.03 | 0.67 | 1.54 | 0.864 | 0.17 | 0.15 | 1.14 | 0.62 | 1.99 | 0.616 |
| Year 1 prior | 0.22 | 0.20 | 1.13 | 0.71 | 1.71 | 0.564 | 0.12 | 0.24 | 0.49 | 0.23 | 0.94 | 0.020 |
| Year 1 post | 0.31 | 0.21 | 1.43 | 0.97 | 2.07 | 0.060 | 0.19 | 0.18 | 1.09 | 0.60 | 1.86 | 0.730 |
| Year 2 post | 0.29 | 0.20 | 1.46 | 0.97 | 2.14 | 0.054 | 0.21 | 0.17 | 1.27 | 0.73 | 2.12 | 0.346 |

OTF-W= Officially Tuberculosis Free Withdrawn (*Mycobacterium bovis* infection confirmed by post-mortem tests), OTF-S= Officially Tuberculosis Free Suspended.

**Table S5.** Number of herds in intervention area central zone during follow-up period that belong to the cohort in existence on the baseline date when culling started.

| Intervention area | Cull year | Herds in cohort mapped to area | Percentage of cohort remaining in area | Herds mapped to area but not in cohort |
| --- | --- | --- | --- | --- |
| Gloucestershire |  |  |  |  |
|  | Year 3 prior | 200 | 93.0 | 36 |
|  | Year 2 prior | 203 | 94.4 | 23 |
|  | Year 1 prior | 208 | 96.7 | 6 |
|  | Year 1 post | 215* | 100.0 | 0 |
|  | Year 2 post | 203 | 94.9 | 7 |
|  | Year 3 post | 178 | 82.8 | 21 |
|  | Year 4 post | 174 | 80.9 | 25 |
| Somerset |  |  |  |  |
|  | Year 3 prior | 136 | 88.3 | 20 |
|  | Year 2 prior | 145 | 94.2 | 11 |
|  | Year 1 prior | 149 | 96.8 | 8 |
|  | Year 1 post | 154* | 100.0 | 0 |
|  | Year 2 post | 147 | 95.5 | 9 |
|  | Year 3 post | 133 | 86.4 | 11 |
|  | Year 4 post | 130 | 84.4 | 22 |
| Dorset |  |  |  |  |
|  | Year 3 prior | 143 | 91.1 | 37 |
|  | Year 2 prior | 146 | 93.0 | 31 |
|  | Year 1 prior | 149 | 94.9 | 16 |
|  | Year 1 post | 157* | 100.0 | 0 |
|  | Year 2 post | 151 | 96.2 | 7 |

*Herds in the intervention area central zone on the baseline date when culling started.

**Table S6.** Effects in the central zones after removal of individual comparison areas from final models (Table 4, model A; Table 5, model C).

|  | Gloucestershire | | | Somerset | | | Dorset | | | Goodness of fit p values | |
| --- | --- | --- | --- | --- | --- | --- | --- | --- | --- | --- | --- |
| Model | IRR | 95% CI | | IRR | 95% CI | | IRR | 95% CI | | Deviance | Pearson |
| Models of effects from four years culling with 2 intervention and 18 comparison areas (Table 4, model A) | | | | | | | | | | | |
| Final model | 0.34 | 0.29 | 0.39 | 0.63 | 0.58 | 0.69 | NA | NA | NA | 0.628 | 0.637 |
| Comparison area removed | | | | | | |  |  |  |  |  |
| WG01 | 0.33 | 0.29 | 0.39 | 0.64 | 0.60 | 0.69 | NA | NA | NA | 0.687 | 0.691 |
| WG02 | 0.34 | 0.30 | 0.39 | 0.63 | 0.58 | 0.69 | NA | NA | NA | 0.540 | 0.549 |
| WG03 | 0.39 | 0.36 | 0.43 | 0.62 | 0.57 | 0.69 | NA | NA | NA | 0.910 | 0.911 |
| WG04 | 0.34 | 0.29 | 0.39 | 0.63 | 0.58 | 0.69 | NA | NA | NA | 0.539 | 0.548 |
| WG05 | 0.32 | 0.29 | 0.36 | 0.64 | 0.60 | 0.69 | NA | NA | NA | 0.824 | 0.829 |
| WG06 | 0.34 | 0.29 | 0.39 | 0.63 | 0.58 | 0.68 | NA | NA | NA | 0.542 | 0.549 |
| WG07 | 0.34 | 0.30 | 0.39 | 0.64 | 0.59 | 0.69 | NA | NA | NA | 0.606 | 0.618 |
| WG08 | 0.32 | 0.28 | 0.37 | 0.60 | 0.55 | 0.66 | NA | NA | NA | 0.731 | 0.739 |
| WG10 | 0.34 | 0.29 | 0.40 | 0.63 | 0.58 | 0.70 | NA | NA | NA | 0.539 | 0.548 |
| WS01 | 0.34 | 0.30 | 0.39 | 0.60 | 0.55 | 0.64 | NA | NA | NA | 0.826 | 0.823 |
| WS02 | 0.35 | 0.29 | 0.41 | 0.67 | 0.56 | 0.80 | NA | NA | NA | 0.564 | 0.571 |
| WS03 | 0.35 | 0.29 | 0.41 | 0.64 | 0.58 | 0.71 | NA | NA | NA | 0.550 | 0.555 |
| WS04 | 0.34 | 0.29 | 0.39 | 0.63 | 0.58 | 0.68 | NA | NA | NA | 0.539 | 0.548 |
| WS05 | 0.36 | 0.31 | 0.41 | 0.66 | 0.59 | 0.74 | NA | NA | NA | 0.636 | 0.647 |
| WS07 | 0.34 | 0.29 | 0.39 | 0.63 | 0.57 | 0.69 | NA | NA | NA | 0.566 | 0.576 |
| WS08 | 0.33 | 0.28 | 0.38 | 0.63 | 0.59 | 0.67 | NA | NA | NA | 0.641 | 0.647 |
| WS09 | 0.34 | 0.29 | 0.40 | 0.63 | 0.58 | 0.68 | NA | NA | NA | 0.540 | 0.548 |
| WS10 | 0.35 | 0.30 | 0.39 | 0.63 | 0.58 | 0.68 | NA | NA | NA | 0.691 | 0.703 |
| Model of effects from two years culling with 3 intervention and 26 comparison areas (Table 5, model C) | | | | | | | | | | | |
| Final model | 0.84 | 0.74 | 0.95 | 0.79 | 0.67 | 0.93 | 1.10 | 0.96 | 1.27 | 0.529 | 0.562 |
| Comparison area removed | | | | | | |  |  |  |  |  |
| WG01 | 0.86 | 0.76 | 0.97 | 0.80 | 0.67 | 0.94 | 1.14 | 0.97 | 1.34 | 0.538 | 0.566 |
| WG02 | 0.84 | 0.74 | 0.95 | 0.79 | 0.67 | 0.93 | 1.10 | 0.96 | 1.27 | 0.459 | 0.492 |
| WG03 | 0.86 | 0.76 | 0.98 | 0.77 | 0.66 | 0.90 | 1.05 | 0.91 | 1.20 | 0.679 | 0.707 |
| WG04 | 0.82 | 0.71 | 0.95 | 0.78 | 0.66 | 0.92 | 1.10 | 0.95 | 1.27 | 0.465 | 0.497 |
| WG05 | 0.84 | 0.74 | 0.95 | 0.79 | 0.68 | 0.92 | 1.10 | 0.95 | 1.28 | 0.458 | 0.491 |
| WG06 | 0.79 | 0.67 | 0.93 | 0.79 | 0.67 | 0.93 | 1.10 | 0.96 | 1.27 | 0.458 | 0.491 |
| WG07 | 0.81 | 0.72 | 0.91 | 0.81 | 0.68 | 0.95 | 1.08 | 0.95 | 1.23 | 0.616 | 0.644 |
| WG08 | 0.81 | 0.72 | 0.91 | 0.75 | 0.65 | 0.87 | 1.08 | 0.97 | 1.20 | 0.715 | 0.743 |
| WG10 | 0.84 | 0.74 | 0.95 | 0.79 | 0.67 | 0.93 | 1.10 | 0.96 | 1.27 | 0.529 | 0.562 |
| WS01 | 0.81 | 0.73 | 0.91 | 0.73 | 0.66 | 0.81 | 1.06 | 0.95 | 1.18 | 0.951 | 0.950 |
| WS02 | 0.85 | 0.73 | 0.98 | 0.80 | 0.64 | 1.01 | 1.10 | 0.96 | 1.28 | 0.461 | 0.492 |
| WS03 | 0.88 | 0.75 | 1.03 | 0.86 | 0.70 | 1.06 | 1.21 | 0.99 | 1.48 | 0.585 | 0.588 |
| WS04 | 0.82 | 0.72 | 0.93 | 0.78 | 0.66 | 0.91 | 1.08 | 0.93 | 1.25 | 0.487 | 0.521 |
| WS05 | 0.83 | 0.74 | 0.94 | 0.81 | 0.68 | 0.96 | 1.10 | 0.96 | 1.26 | 0.651 | 0.672 |
| WS07 | 0.84 | 0.74 | 0.94 | 0.79 | 0.67 | 0.92 | 1.11 | 0.96 | 1.29 | 0.460 | 0.493 |
| WS08 | 0.83 | 0.74 | 0.94 | 0.80 | 0.68 | 0.95 | 1.10 | 0.96 | 1.27 | 0.523 | 0.541 |
| WS09 | 0.82 | 0.70 | 0.96 | 0.82 | 0.70 | 0.96 | 1.13 | 1.00 | 1.27 | 0.567 | 0.605 |
| WS10 | 0.87 | 0.75 | 1.00 | 0.79 | 0.67 | 0.92 | 1.12 | 0.97 | 1.30 | 0.478 | 0.512 |
| DR01 | 0.84 | 0.74 | 0.96 | 0.79 | 0.67 | 0.92 | 1.08 | 0.89 | 1.30 | 0.466 | 0.499 |
| DR02 | 0.84 | 0.75 | 0.95 | 0.80 | 0.68 | 0.93 | 1.11 | 0.96 | 1.29 | 0.463 | 0.496 |
| DR03 | 0.84 | 0.74 | 0.95 | 0.79 | 0.67 | 0.93 | 1.10 | 0.95 | 1.28 | 0.458 | 0.491 |
| DR04 | 0.84 | 0.74 | 0.95 | 0.79 | 0.67 | 0.93 | 1.10 | 0.96 | 1.27 | 0.460 | 0.492 |
| DR06 | 0.85 | 0.75 | 0.97 | 0.81 | 0.68 | 0.96 | 1.09 | 0.95 | 1.25 | 0.477 | 0.513 |
| DR07 | 0.82 | 0.73 | 0.92 | 0.78 | 0.67 | 0.92 | 1.15 | 0.99 | 1.33 | 0.559 | 0.595 |
| DR08 | 0.80 | 0.66 | 0.97 | 0.79 | 0.66 | 0.93 | 1.08 | 0.92 | 1.28 | 0.466 | 0.501 |
| DR10 | 0.85 | 0.74 | 0.97 | 0.79 | 0.67 | 0.93 | 1.10 | 0.95 | 1.27 | 0.465 | 0.499 |

IRR=Incidence rate ratio (incidence rate in the intervention area/incidence rate in comparison areas). Goodness of fit probability values >0.05 indicate model fit is good. Gloucestershire and Somerset intervention areas have had four years of culling and Dorset has had two years of culling. Comparison areas lost because of overlap by newly licenced areas were WG09, WS06, DR05 and DR09.

**Table S7.** Effects in the buffer zones after removal of individual comparison areas from final models (Table 4, model B; Table 5, model D).

|  | Gloucestershire | | | Somerset | | | Dorset | | | Goodness of fit p values | |
| --- | --- | --- | --- | --- | --- | --- | --- | --- | --- | --- | --- |
| Model | IRR | 95% CI | | IRR | 95% CI | | IRR | 95% CI | | Deviance | Pearson |
| Model of effects from four years culling with 2 intervention and 18 comparison areas (Table 4, model B) | | | | | | | | | | | |
| Final model | 0.64 | 0.58 | 0.70 | 0.97 | 0.80 | 1.18 | NA | NA | NA | 0.212 | 0.197 |
| Comparison area removed | | | | | | | | | | | |
| WG01B | 0.64 | 0.58 | 0.70 | 0.97 | 0.80 | 1.18 | NA | NA | NA | 0.160 | 0.147 |
| WG02B | 0.65 | 0.58 | 0.73 | 0.98 | 0.80 | 1.21 | NA | NA | NA | 0.160 | 0.147 |
| WG03B | 0.64 | 0.50 | 0.83 | 0.97 | 0.75 | 1.24 | NA | NA | NA | 0.158 | 0.146 |
| WG04B | 0.64 | 0.58 | 0.71 | 0.94 | 0.79 | 1.14 | NA | NA | NA | 0.179 | 0.165 |
| WG05B | 0.63 | 0.58 | 0.70 | 0.96 | 0.78 | 1.19 | NA | NA | NA | 0.172 | 0.159 |
| WG06B | 0.64 | 0.58 | 0.70 | 1.00 | 0.77 | 1.29 | NA | NA | NA | 0.162 | 0.149 |
| WG07B | 0.63 | 0.58 | 0.69 | 1.01 | 0.83 | 1.23 | NA | NA | NA | 0.221 | 0.213 |
| WG08B | 0.64 | 0.58 | 0.70 | 0.99 | 0.82 | 1.20 | NA | NA | NA | 0.180 | 0.171 |
| WG10B | 0.64 | 0.58 | 0.71 | 0.97 | 0.78 | 1.20 | NA | NA | NA | 0.159 | 0.146 |
| WS01B | 0.64 | 0.59 | 0.71 | 0.94 | 0.80 | 1.10 | NA | NA | NA | 0.550 | 0.557 |
| WS02B | 0.65 | 0.58 | 0.73 | 1.03 | 0.80 | 1.32 | NA | NA | NA | 0.165 | 0.155 |
| WS03B | 0.64 | 0.57 | 0.72 | 0.93 | 0.74 | 1.18 | NA | NA | NA | 0.167 | 0.161 |
| WS04B | 0.64 | 0.57 | 0.71 | 0.97 | 0.80 | 1.18 | NA | NA | NA | 0.159 | 0.148 |
| WS05B | 0.64 | 0.61 | 0.67 | 0.89 | 0.79 | 1.00 | NA | NA | NA | 0.967 | 0.967 |
| WS07B | 0.64 | 0.58 | 0.70 | 0.97 | 0.80 | 1.18 | NA | NA | NA | 0.161 | 0.149 |
| WS08B | 0.61 | 0.54 | 0.69 | 1.05 | 0.86 | 1.27 | NA | NA | NA | 0.360 | 0.344 |
| WS09B | 0.67 | 0.60 | 0.76 | 0.91 | 0.76 | 1.08 | NA | NA | NA | 0.230 | 0.221 |
| WS10B | 0.61 | 0.56 | 0.68 | 1.07 | 0.87 | 1.30 | NA | NA | NA | 0.542 | 0.549 |
| Model with effects from two years culling with 3 intervention and 26 comparison areas (Table 5, model D) | | | | | | | | | | | |
| Final model | 0.89 | 0.66 | 1.20 | 1.09 | 0.91 | 1.30 | 0.45 | 0.37 | 0.54 | 0.707 | 0.600 |
| Comparison area removed | | | | | | | | | | | |
| WG01B | 0.87 | 0.66 | 1.16 | 1.12 | 0.93 | 1.35 | 0.45 | 0.37 | 0.54 | 0.684 | 0.578 |
| WG02B | 0.94 | 0.69 | 1.27 | 1.12 | 0.93 | 1.34 | 0.45 | 0.37 | 0.54 | 0.687 | 0.578 |
| WG03B | 1.32 | 0.89 | 1.96 | 0.93 | 0.75 | 1.15 | 0.44 | 0.38 | 0.52 | 0.787 | 0.718 |
| WG04B | 0.77 | 0.55 | 1.08 | 1.12 | 0.96 | 1.32 | 0.45 | 0.37 | 0.54 | 0.728 | 0.657 |
| WG05B | 0.94 | 0.70 | 1.28 | 1.09 | 0.92 | 1.30 | 0.44 | 0.36 | 0.53 | 0.655 | 0.542 |
| WG06B | 0.83 | 0.63 | 1.08 | 1.05 | 0.87 | 1.25 | 0.46 | 0.38 | 0.55 | 0.675 | 0.568 |
| WG07B | 0.89 | 0.66 | 1.20 | 1.08 | 0.89 | 1.31 | 0.45 | 0.37 | 0.54 | 0.648 | 0.534 |
| WG08B | 0.89 | 0.65 | 1.22 | 1.06 | 0.89 | 1.26 | 0.44 | 0.37 | 0.53 | 0.701 | 0.595 |
| WG10B | 0.90 | 0.66 | 1.22 | 1.09 | 0.91 | 1.30 | 0.46 | 0.38 | 0.55 | 0.671 | 0.564 |
| WS01B | 0.91 | 0.66 | 1.24 | 1.06 | 0.87 | 1.29 | 0.44 | 0.37 | 0.53 | 0.659 | 0.544 |
| WS02B | 0.90 | 0.65 | 1.22 | 1.08 | 0.87 | 1.33 | 0.45 | 0.37 | 0.55 | 0.646 | 0.534 |
| WS03B | 0.88 | 0.66 | 1.19 | 1.12 | 0.89 | 1.40 | 0.46 | 0.37 | 0.57 | 0.655 | 0.549 |
| WS04B | 0.90 | 0.67 | 1.21 | 1.09 | 0.91 | 1.30 | 0.45 | 0.37 | 0.54 | 0.658 | 0.555 |
| WS05B | 0.91 | 0.69 | 1.20 | 1.14 | 0.95 | 1.36 | 0.45 | 0.37 | 0.55 | 0.758 | 0.673 |
| WS07B | 0.87 | 0.63 | 1.22 | 1.09 | 0.91 | 1.31 | 0.46 | 0.38 | 0.55 | 0.678 | 0.588 |
| WS08B | 0.82 | 0.56 | 1.20 | 1.07 | 0.90 | 1.28 | 0.45 | 0.37 | 0.54 | 0.665 | 0.561 |
| WS09B | 0.93 | 0.69 | 1.26 | 1.11 | 0.93 | 1.32 | 0.47 | 0.38 | 0.57 | 0.681 | 0.580 |
| WS10B | 0.88 | 0.66 | 1.17 | 1.10 | 0.92 | 1.31 | 0.45 | 0.37 | 0.55 | 0.654 | 0.536 |
| DR01B | 0.90 | 0.67 | 1.20 | 1.09 | 0.92 | 1.31 | 0.46 | 0.37 | 0.56 | 0.658 | 0.530 |
| DR02B | 0.89 | 0.66 | 1.21 | 1.09 | 0.90 | 1.31 | 0.45 | 0.35 | 0.56 | 0.645 | 0.534 |
| DR03B | 0.88 | 0.68 | 1.14 | 1.16 | 1.00 | 1.34 | 0.41 | 0.33 | 0.50 | 0.772 | 0.674 |
| DR04B | 0.90 | 0.71 | 1.13 | 1.09 | 0.91 | 1.31 | 0.46 | 0.38 | 0.54 | 0.967 | 0.965 |
| DR06B | 0.87 | 0.65 | 1.17 | 1.03 | 0.87 | 1.21 | 0.45 | 0.38 | 0.54 | 0.726 | 0.577 |
| DR07B | 0.89 | 0.66 | 1.20 | 1.09 | 0.90 | 1.33 | 0.45 | 0.37 | 0.53 | 0.646 | 0.529 |
| DR08B | 0.89 | 0.66 | 1.20 | 1.09 | 0.91 | 1.30 | 0.45 | 0.35 | 0.57 | 0.645 | 0.533 |
| DR10B | 0.86 | 0.64 | 1.16 | 1.06 | 0.89 | 1.26 | 0.41 | 0.35 | 0.48 | 0.820 | 0.742 |

IRR=Incidence rate ratio (incidence rate in the intervention area/incidence rate in comparison areas). Goodness of fit probability values >0.05 indicate model fit is good. Gloucestershire and Somerset intervention areas have had four years of culling and Dorset has had two years of culling. Comparison areas lost because of overlap with newly licenced areas were WG09, WS06, DR05 and DR09.

**Table S8.** Multivariable Poisson regression models of the association between all TB incidence rates and four years culling in Gloucestershire and Somerset.

|  | IRR | Robust SE | p value | 95% Confidence interval | |
| --- | --- | --- | --- | --- | --- |
| **Model E Central zones of Somerset and Gloucestershire** |  |  |  |  |  |
| **Intervention effect in Somerset area** | 0.69 | 0.02 | <0.001 | 0.64 | 0.74 |
| **Intervention effect in Gloucestershire** | 0.43 | 0.03 | <0.001 | 0.38 | 0.49 |
| Area=Somerset | 1.03 | 0.05 | 0.560 | 0.94 | 1.13 |
| Log transformed herd years at risk for 4 years of culling | 3.85 | 0.42 | <0.001 | 3.11 | 4.76 |
| Log transformed TB incidence rate over 3 years prior | 1.23 | 0.08 | 0.001 | 1.09 | 1.40 |
| Log transformed median herd size | 1.23 | 0.32 | 0.426 | 0.74 | 2.07 |
| Percentage of herds that were dairy | 1.01 | 0.00 | <0.001 | 1.01 | 1.02 |
| Distance to intervention (km) | 1.00 | 0.00 | <0.001 | 1.00 | 1.00 |
| Log transformed number of badgers culled historically | 1.04 | 0.01 | <0.001 | 1.02 | 1.05 |
| Percentage of farms with at least 1 land parcel in zone | 1.01 | 0.01 | 0.148 | 1.00 | 1.02 |
| **Model F Buffer zones of Somerset and Gloucestershire** |  |  |  |  |  |
| **Intervention effect in Somerset area** | 1.13 | 0.10 | 0.178 | 0.94 | 1.36 |
| **Intervention effect in Gloucestershire area** | 0.85 | 0.05 | 0.005 | 0.76 | 0.95 |
| Area=Somerset | 1.15 | 0.06 | 0.011 | 1.03 | 1.28 |
| Log transformed herd years at risk for 4 years of culling | 3.31 | 0.39 | <0.001 | 2.63 | 4.17 |
| Log transformed TB incidence rate over 3 years prior | 1.38 | 0.14 | 0.002 | 1.12 | 1.69 |
| Log transformed median herd size | 1.40 | 0.14 | 0.001 | 1.15 | 1.69 |
| Percentage of land classed as urban | 1.04 | 0.00 | <0.001 | 1.03 | 1.05 |
| Log transformed number of badgers culled historically | 1.01 | 0.02 | 0.513 | 0.98 | 1.04 |
| Percentage of farms with all land in zone | 1.01 | 0.01 | 0.043 | 1.00 | 1.03 |

TB= bovine TB incidence rate in cattle herds which includes OTF-W (Officially Tuberculosis Free status Withdrawn) plus OTF-S (Officially Tuberculosis Fee status Suspended) incidents. IRR=Incidence Rate Ratio. Intervention is industry-led culling. SE= standard error. Observations = 20 in both models. Deviance goodness of fit (gof) p values = 0.708 and 0.519, Pearson gof p values=0.698 and 0.529 for models A and B respectively.

**Table S9.** Multivariable Poisson regression models of the association between all TB incidence rates and two years culling in Gloucestershire, Somerset and Dorset.

|  | IRR | Robust SE | p value | 95% Confidence interval | |
| --- | --- | --- | --- | --- | --- |
| **Model G Central zones of Somerset, Gloucestershire and Dorset** | |  |  |  |  |
| **Intervention effect in Somerset area** | 0.85 | 0.08 | 0.078 | 0.71 | 1.02 |
| **Intervention effect in Gloucestershire** | 0.90 | 0.07 | 0.171 | 0.78 | 1.05 |
| **Intervention effect in Dorset area** | 1.32 | 0.12 | 0.002 | 1.10 | 1.58 |
| Area=Somerset | 0.94 | 0.09 | 0.496 | 0.79 | 1.12 |
| Area=Dorset | 0.60 | 0.22 | 0.167 | 0.30 | 1.23 |
| Log transformed herd years at risk for 2 years of culling | 1.99 | 0.24 | <0.001 | 1.58 | 2.51 |
| Log transformed OTF-W incidence rate over 3 years prior | 1.42 | 0.17 | 0.003 | 1.13 | 1.78 |
| Log transformed median herd size | 1.94 | 0.94 | 0.169 | 0.75 | 4.99 |
| Percentage of land in flood zone 3 | 0.99 | 0.00 | <0.001 | 0.98 | 0.99 |
| Distance to intervention (km) | 1.00 | 0.00 | 0.319 | 1.00 | 1.00 |
| Between 0 and 1 badgers removed 1972-2006 | Badgers removed reference category | | | |  |
| Between 2 and 35 badgers removed 1972-2006 | 0.96 | 0.09 | 0.653 | 0.79 | 1.16 |
| Between 38 and 72 badgers removed 1972-2006 | 0.91 | 0.07 | 0.242 | 0.78 | 1.06 |
| Between 79 and 356 badgers removed 1972-2006 | 1.37 | 0.18 | 0.014 | 1.07 | 1.77 |
| Between 371 and 1589 badgers removed 1972-2006 | 0.95 | 0.07 | 0.510 | 0.81 | 1.11 |
| **Model H Buffer zones of Somerset, Gloucestershire and Dorset** | |  |  |  |  |
| **Intervention effect in Somerset area** | 1.08 | 0.08 | 0.317 | 0.93 | 1.25 |
| **Intervention effect in Gloucestershire** | 1.06 | 0.10 | 0.546 | 0.88 | 1.28 |
| **Intervention effect Dorset** | 0.91 | 0.08 | 0.298 | 0.77 | 1.08 |
| Area=Somerset | 1.07 | 0.07 | 0.279 | 0.95 | 1.22 |
| Area=Dorset | 0.92 | 0.08 | 0.354 | 0.78 | 1.09 |
| Log transformed herd years at risk for 2 years of culling | 2.61 | 0.22 | <0.001 | 2.21 | 3.07 |
| Log transformed OTF-W incidence rate over 3 years prior | 1.55 | 0.08 | <0.001 | 1.40 | 1.70 |
| Log transformed median herd size | 1.21 | 0.10 | 0.022 | 1.03 | 1.43 |
| Percentage of land classed as urban | 1.05 | 0.01 | <0.001 | 1.04 | 1.07 |
| Length of motorway (km) | 1.00 | <0.001 | 0.004 | 1.00 | 1.00 |
| Distance to intervention (km) | 1.00 | <0.01 | 0.047 | 0.99 | 1.00 |
| Percentage of farms with all land in central or buffer zones | 1.01 | <0.01 | 0.002 | 1.00 | 1.02 |

TB= bovine TB incidence rate in cattle herds which includes OTF-W (Officially Tuberculosis Free status Withdrawn) plus OTF-S (Officially Tuberculosis Fee status Suspended) incidents. IRR=Incidence Rate Ratio. Intervention is industry-led culling. SE= Standard error. Observations = 29 in both models. Deviance goodness of fit (gof) p values were 0.193 and 0.783 and Pearson gof p values were 0.182 and 0.723 respectively for models C and D.

**Table S10.** Multivariable Poisson regression model showing the association between OTFW incidence rates and two years culling in Gloucestershire and Somerset, based on Table 4 in Brunton et al. 2017.

|  | IRR | Robust SE | *p* value | 95% Confidence interval | |
| --- | --- | --- | --- | --- | --- |
| **Model I - Central zones (22 observations)** |  |  |  |  |  |
| Intervention effect in Somerset area | 0.79 | 0.08 | 0.017 | 0.65 | 0.96 |
| Intervention effect in Gloucestershire area | 0.47 | 0.03 | <0.001 | 0.40 | 0.54 |
| Area=Somerset | 1.13 | 0.27 | 0.602 | 0.71 | 1.81 |
| Log transformed herds years at risk in first 2 years of culling | 3.92 | 0.87 | <0.001 | 2.54 | 6.05 |
| Log transformed OTFW incidence rate over 3 years prior | 1.16 | 0.25 | 0.493 | 0.76 | 1.76 |
| Log transformed median herd size | 0.61 | 0.62 | 0.628 | 0.08 | 4.52 |
| Percentage of herd that are dairy | 1.01 | <0.01 | <0.001 | 1.00 | 1.02 |
| Distance to intervention (km) | 1.00 | <0.01 | <0.001 | 1.00 | 1.00 |
| Estimated badger density per 100 km^2^ | 0.98 | 0.02 | 0.219 | 0.95 | 1.01 |
| Percentage of land involved in proactive culling in the RBCT | 1.06 | 0.04 | 0.099 | 0.99 | 1.13 |
| Log transformed number of badgers culled historically | 1.03 | 0.01 | 0.032 | 1.00 | 1.06 |
| Percent. of farms with more than one fragment of land in the area | 1.03 | 0.01 | <0.001 | 1.02 | 1.05 |
| **Model J - Buffer areas (22 observations)** |  |  |  |  |  |
| Intervention effect in Somerset area | 1.09 | 0.23 | 0.683 | 0.72 | 1.65 |
| Intervention effect in Gloucestershire area | 1.01 | 0.13 | 0.917 | 0.78 | 1.31 |
| Area=Somerset | 1.06 | 0.12 | 0.571 | 0.86 | 1.32 |
| Log transformed herd years at risk in first 2 years of culling | 2.15 | 0.26 | <0.001 | 1.71 | 2.72 |
| Log transformed OTF-W incidence rate over 3 years prior | 1.78 | 0.11 | <0.001 | 1.58 | 2.01 |
| Log transformed median herd size | 1.04 | 0.14 | 0.737 | 0.81 | 1.35 |
| Percentage of herds that are dairy | 1.01 | 0.01 | 0.071 | 1.00 | 1.02 |
| Distance to intervention (km) | 1.00 | <0.01 | 0.867 | 1.00 | 1.00 |
| Estimated badger density per 100 km^2^ | 1.08 | 0.04 | 0.035 | 1.01 | 1.16 |
| Percentage of land involved in proactive culling in the RBCT | 1.01 | 0.01 | 0.418 | 0.99 | 1.04 |
| Percentage of land classed as urban | 1.02 | 0.02 | 0.303 | 0.99 | 1.05 |
| Percent. of farms with more than one fragment of land in the area | 1.02 | 0.01 | 0.006 | 1.01 | 1.03 |

OTFW (Officially Tuberculosis Free status Withdrawn (*Mycobacterium bovis* infection confirmed by post-mortem tests)). IRR=Incidence Rate Ratio. Intervention=industry-led culling. Deviance goodness of fit (gof) p values = 0.176 and 0.739, Pearson gof p values=0.201 and 0.745 for models I and J respectively. Covariates in the above model are the same as in Brunton et al. 2017 except herd years at risk, percentage of land in a Randomised Badger Culling Trial (RBCT) proactive area have been revised following data cleansing for the most recent analysis. Badger sett density variable has been replaced by Badger density. Estimates for effects following two years culling in Brunton et al. 2017 were 0.41 (95%CI 0.34, 0.51) and 0.79 (95% CI 0.72, 087) for the central zones and 0.91 (95% CI 0.77, 1.07) and 1.38 (95% CI 1.10, 1.75) and for the buffer zones for Gloucestershire and Somerset respectively.

Reference

Brunton, L.A. et al. Assessing the effects of the first 2 years of industry-led badger culling in England on the incidence of bovine tuberculosis in cattle in 2013–2015. Ecol Evol. 7, 7213-7230 (2017).

**Table S11.** Multivariable Poisson regression models of the association between OTF-W incidence rates and four years culling in Gloucestershire and Somerset *(without robust standard errors)*.

|  | IRR | SE | p value | 95% Confidence interval | |
| --- | --- | --- | --- | --- | --- |
| **Model K Central zones of Somerset and Gloucestershire** |  |  |  |  |  |
| **Intervention effect in Somerset area** | 0.63 | 0.09 | 0.002 | 0.48 | 0.84 |
| **Intervention effect in Gloucestershire** | 0.34 | 0.06 | <0.001 | 0.25 | 0.47 |
| Area=Somerset | 1.10 | 0.09 | 0.249 | 0.94 | 1.29 |
| Log transformed herd years at risk for 4 years of culling | 4.26 | 0.69 | <0.001 | 3.11 | 5.85 |
| Log transformed OTF-W incidence rate over 3 years prior | 1.26 | 0.12 | 0.018 | 1.04 | 1.53 |
| Log transformed median herd size | 0.86 | 0.34 | 0.695 | 0.39 | 1.86 |
| Percentage of herds that were dairy | 1.01 | 0.01 | 0.023 | 1.00 | 1.02 |
| Distance to intervention (km) | 1.00 | <0.01 | <0.001 | 0.99 | 1.00 |
| Log transformed number of badgers culled historically | 1.04 | 0.01 | 0.002 | 1.01 | 1.06 |
| Percentage of farms with at least 1 land parcel in area | 1.02 | 0.01 | 0.050 | 1.00 | 1.03 |
| **Model L Buffer zones of Somerset and Gloucestershire** |  |  |  |  |  |
| **Intervention effect in Somerset area** | 0.97 | 0.20 | 0.891 | 0.64 | 1.47 |
| **Intervention effect in Gloucestershire area** | 0.64 | 0.12 | 0.021 | 0.44 | 0.93 |
| Area=Somerset | 1.22 | 0.11 | 0.025 | 1.03 | 1.46 |
| Log transformed herd years at risk for 4 years of culling | 3.28 | 0.51 | <0.001 | 2.42 | 4.46 |
| Log transformed OTF-W incidence rate over 3 years prior | 1.17 | 0.09 | 0.031 | 1.01 | 1.35 |
| Log transformed median herd size | 1.39 | 0.25 | 0.062 | 0.98 | 1.97 |
| Percentage of land classed as urban | 1.04 | 0.01 | <0.001 | 1.03 | 1.06 |
| Log transformed number of badgers culled historically | 1.04 | 0.02 | 0.057 | 1.00 | 1.07 |
| Percentage of farms with all land inside area | 1.02 | 0.01 | 0.100 | 1.00 | 1.04 |

OTF-W = Officially Tuberculosis Free status Withdrawn. IRR=Incidence Rate Ratio. Intervention is industry-led culling. SE= standard error. Observations = 20 in both models. Deviance goodness of fit (gof) p values = 0.444 and 0.113, Pearson gof p values=0.453 and 0.103 for models K and L respectively.

**Table S12.** Multivariable Poisson regression models of the association between OTF-W incidence rates and two years culling in Gloucestershire, Somerset and Dorset (*without robust standard errors)*.

|  | IRR | SE | p value | 95% Confidence interval | |
| --- | --- | --- | --- | --- | --- |
| **Model M Central zones of Somerset, Gloucestershire and Dorset** | |  |  |  |  |
| **Intervention effect in Somerset area** | 0.79 | 0.15 | 0.212 | 0.55 | 1.14 |
| **Intervention effect in Gloucestershire** | 0.84 | 0.18 | 0.400 | 0.55 | 1.27 |
| **Intervention effect in Dorset area** | 1.10 | 0.20 | 0.592 | 0.77 | 1.58 |
| Area=Somerset | 1.01 | 0.10 | 0.913 | 0.83 | 1.22 |
| Area=Dorset | 0.69 | 0.23 | 0.251 | 0.36 | 1.31 |
| Log transformed herd years at risk for 2 years of culling | 2.34 | 0.38 | <0.001 | 1.70 | 3.22 |
| Log transformed OTF-W incidence rate over 3 years prior | 1.59 | 0.20 | <0.001 | 1.24 | 2.05 |
| Log transformed median herd size | 1.49 | 0.62 | 0.344 | 0.65 | 3.39 |
| Percentage of land in flood zone 3 | 0.98 | 0.01 | <0.001 | 0.97 | 0.99 |
| Distance to intervention (km) | 1.00 | 0.00 | 0.060 | 0.99 | 1.00 |
| Between 0 and 1 badgers removed 1972-2006 | Badgers removed reference category | | | |  |
| Between 2 and 35 badgers removed 1972-2006 | 0.99 | 0.10 | 0.895 | 0.81 | 1.21 |
| Between 38 and 72 badgers removed 1972-2006 | 0.89 | 0.10 | 0.311 | 0.70 | 1.12 |
| Between 79 and 356 badgers removed 1972-2006 | 1.41 | 0.19 | 0.012 | 1.08 | 1.84 |
| Between 371 and 1589 badgers removed 1972-2006 | 0.91 | 0.10 | 0.386 | 0.72 | 1.13 |
| **Model N Buffer zones of Somerset, Gloucestershire and Dorset** | |  |  |  |  |
| **Intervention effect in Somerset area** | 1.09 | 0.28 | 0.744 | 0.66 | 1.80 |
| **Intervention effect in Gloucestershire** | 0.89 | 0.30 | 0.727 | 0.46 | 1.71 |
| **Intervention effect Dorset** | 0.45 | 0.14 | 0.010 | 0.24 | 0.82 |
| Area=Somerset | 1.09 | 0.13 | 0.458 | 0.87 | 1.38 |
| Area=Dorset | 0.80 | 0.12 | 0.151 | 0.59 | 1.09 |
| Log transformed herd years at risk for 2 years of culling | 2.45 | 0.30 | <0.001 | 1.92 | 3.13 |
| Log transformed OTF-W incidence rate over 3 years prior | 1.33 | 0.11 | <0.001 | 1.14 | 1.56 |
| Log transformed median herd size | 1.32 | 0.27 | 0.187 | 0.88 | 1.98 |
| Percentage of land classed as urban | 1.05 | 0.02 | <0.001 | 1.02 | 1.08 |
| Length of motorway (km) | 0.99 | 0.01 | 0.332 | 0.96 | 1.01 |
| Distance to intervention (km) | 1.00 | <0.01 | 0.107 | 0.99 | 1.03 |
| Percentage of land inside central or buffer areas | 1.01 | 0.01 | 0.071 | 1.00 | 1.02 |

OTF-W= Officially Tuberculosis Free status Withdrawn. IRR=Incidence Rate Ratio. Intervention is industry-led culling. SE= Standard error. Observations = 29 in both models. Deviance goodness of fit (gof) p values were 0.318 and 0.506 and Pearson gof p values were 0.347 and 0.395 respectively for models M and N.

**Table S13. Estimates of the proportion of badgers removed in year 1 in the intervention areas of Somerset, Gloucestershire and Dorset**

| Area | Cull year | Year | Land area (km^2^) | Badger population at baseline | Population density (badgers/km^2^) before removal (95% CI) | Number of badgers removed | Population density (badgers/km^2^) after removal (95% CI) | Percentage of baseline population removed |
| --- | --- | --- | --- | --- | --- | --- | --- | --- |
| Gloucestershire | 1 | 2013 | 311 | 1617 | 5.2 (5.1 to 5.3) | 924 | 2.2 (2.1 to 2.3) | 57.1 |
| Somerset | 1 | 2013 | 256 | 1178 | 4.6 (4.5 to 4.7) | 955 | 0.9 (0.6 to 1.2) | 81.1 |
| Dorset | 1 | 2015 | 223 | 2163 | 9.7 (9.6 to 9.8) | 756 | 6.3 (6.2 to 6.4) | 34.9 |

Badger population at baseline is estimated from land area licenced for culling and badger density based on local sett survey data (see supplement page 2). Numbers of badger removed through culling are from AHVLA 2014 and Defra 2015.

References

Animal Health and Veterinary Laboratories Agency (AHVLA). The efficacy of badger population reduction by controlled shooting and cage trapping, and the change in badger activity following culling from 27/08/2013 to 28/11/2013. Report to Defra. 6 February 2014. Preprint at

<https://assets.publishing.service.gov.uk/government/uploads/system/uploads/attachment_data/file/300385/ahvla-extension-efficacy.pdf> (2014)

Department for Environment, Food & Rural Affairs (Defra). Summary of badger control monitoring during 2015. December 2015. Preprint at

<https://assets.publishing.service.gov.uk/government/uploads/system/uploads/attachment_data/file/486604/summary-badger-control-monitoring-2015.pdf> (2015)
